# N2SIMBA: from Network topology to SIMulation of interactions and BActerial abundance, using microbial consumer resource model

**DOI:** 10.3389/fbinf.2026.1783447

**Published:** 2026-05-20

**Authors:** Matteo Baldan, Giacomo Baruzzo, Piero Mariotto, Ada Rossato, Marco Cappellato, Barbara Di Camillo

**Affiliations:** 1 Department of Information Engineering, University of Padua, Padova, Italy; 2 Department of Comparative Biomedicine Food Science, University of Padua, Padova, Italy; 3 Padua Center of Network Medicine, University of Padua, Padova, Italy

**Keywords:** 16 rDNA-seq, computational biology, dynamical systems, ecological networks, microbial communities, numerical simulations

## Abstract

Microorganisms often coexist and establish complex interaction patterns within their environment. Recent advances in efficient and cost-effective 16S rDNA sequencing have greatly improved our ability to characterize microbial communities and to infer networks of interactions among their members. However, validating microbial interaction inference methods remains challenging, as a true and experimentally accessible reference for microbial interactions is generally unavailable, expecially for large networks. For this reason, *in silico* approaches are essential to generate realistic synthetic data with known interaction structures that can serve as gold standards. Here, we introduce N2SIMBA, a modular simulation framework for bacterial communities that takes as input weighted and directed interaction networks, interpreted as known ground-truth microbial interactions, and generates realistic 16S rDNA sequencing count tables as output. N2SIMBA models the dynamics of the microbial community using a consumer–resource framework, in which microbial interactions are mediated by metabolites. Each edge in the input network encodes metabolite-mediated interactions in which microbial species affect one another through the consumption and transformation of shared resources, giving rise to both cooperative and competitive effects. These processes are governed by parameters that describe consumer preferences and metabolic transformation rules, allowing the simulation of biologically plausible interaction mechanisms. Finally, to produce synthetic sequencing data, N2SIMBA simulates the experimental sequencing process by introducing sampling variability and compositional effects, thus generating count tables that resemble real 16S rDNA sequencing datasets. We demonstrate that N2SIMBA enables the generation of realistic *in silico* microbial count tables with known interaction structures, making it possible to systematically evaluate and benchmark microbial network inference methods. As a proof of concept, we compare two widely used interaction inference approaches, showing how N2SIMBA can be used to assess their ability to recover known interactions. In general, N2SIMBA provides a flexible framework for simulating microbial communities and supports the development, testing, and validation of microbial interaction inference methodologies, contributing to more robust interpretations of microbial community data.

## 1 Introduction

The study of bacterial interaction networks is of paramount importance in microbiology and systems biology. These complex ecosystems play a central role in various biological processes that impact human health, environmental dynamics, and agricultural systems (Tom et al., 2003; Hacquard et al., 2015; Sakkos et al., 2023). Although numerous papers addressing the challenges of network reconstruction or reverse engineering (Faust and Raes, 2012a) can be found in the literature, a noticeable gap persists. Despite the wealth of research, a definitive gold standard for assessing and benchmarking such network reconstruction methods remains elusive. Notably, bacterial networks deemed “known” are often of modest scale and are only partially characterized. In terms of *in silico* data, to the best of the authors’ knowledge, there is no simulator for 16S rDNA-seq count tables that accepts a known bacterial interactions network as input. In the literature, there are various approaches to simulate bacterial communities (Srinivasan et al., 2024), but none accepts a bacteria interaction network as input to model the evolution of the bacteria community and are notably not designed to model 16S rDNA-seq count data.

Typically, benchmarking is performed within reverse engineering methods’ articles. These studies simulate the network as a covariance matrix, generating bacterial abundance data and possibly sequencing count data to mimic the availability of 16S or metagenomic data. However, it is crucial to acknowledge that bacterial networks represent primarily indirect interactions. With the exception of instances such as phagocytosis, interactions are mediated by the presence of metabolites that are utilized and produced by bacteria and/or available in the environment.

In this work, we adopt a mathematical approach to model the ecological dynamics of microbial communities. Our chosen method involves using the Microbial Consumer Resource Model (MiCRM) (Marsland et al., 2020b) to represent interactions within the microbial community in a given environment. Our objective is to define a simulation procedure of 16s rDNA-seq count tables that can be used, among other purposes, for benchmarking network reconstruction methods. The interactions within the microbial community are interpreted starting from the metabolic consumption network, and the sequencing step is simulated on *in silico* abundance data of the MiCRM when it reaches steady state conditions.

MiCRM is a variant of generalized consumer-resource models, based on the original formulation of (MacArthur, 1970). MiCRM is a state-space model of Ordinary Differential Equations (ODEs) used to study interactions between bacteria in an ecosystem that describes taxa abundances and resource concentrations as state variables related by first-order differential equations. This model focuses on the dynamics between consumers (i.e., bacteria), which utilize and produce resources, and the resources themselves, which can be substances such as nutrients or other energy sources. Therefore, the abundance of taxa and the concentration of resources represent the entire state of the microbial community described by the MiCRM at any given time (Marsland et al., 2019). The model seeks to understand how the abundance of consumers influences the availability and utilization of resources and *vice versa*. It aims at modeling how an equilibrium or a certain type of dynamic stability is reached between these two elements. As mentioned above, interactions between bacteria are often mediated by the presence of metabolites and chemical substances in the environment. MiCRM seeks to incorporate these indirect dynamics into its simulations (Marsland et al., 2020a). It may consider the feedback and cycles that can emerge within the bacterial ecosystem. For example, an increase in the consumer population may affect resource availability, which in turn can influence the consumer population. Moreover, the design of its model parameters can be adapted to different types of bacterial ecosystems and various experimental conditions (van den Berg et al., 2022).

16S count data simulators aim at generating synthetic 16S rDNA-seq count tables that, starting from some users’ parameters, realistically resemble both the biological variability typical of bacteria communities and the technical noise introduced by sequencing experiments. The simulation parameters can typically be specified by users, estimated from a real 16S count table, or taken from predefined sets of input parameters included in the simulators (Ma et al., 2021a; Patuzzi et al., 2019), offering a wide range of usage modes. Sequencing count data are notoriously hard to model and simulate (Cao et al., 2021; Baruzzo et al., 2019; Zappia et al., 2017) due to their peculiar biological characteristics (e.g., strongly skewed distribution of bacteria abundances, non-independence of taxa abundance, and gene copy number variation) and the noise/biases introduced by the sequencing process (e.g., sparsity, compositionality, uneven sequencing depth, and amplification bias).

The simulation of count data can be classified into two approaches, those who model both biological variability and technical variability according to known biases and forms of error, and those who try to model 16S rDNA-seq data with black box models such as generative adversarial networks (GAN) or other deep learning approaches (Sankaran et al., 2025). The first approach includes methodologies, such as SparseDossa2 (Ma et al., 2021b), metaSPARSim (Patuzzi et al., 2019), MIDASim (He et al., 2024), which use template data to estimate the effect of biological biases, trying to fit model parameters over real data. These methodologies do not model directly specific known biases, such as the copy number variation, for instance, but model the sparsity of the data and the abundance or proportion of present taxa in each sample. Modeling all biological biases requires higher complexity; therefore, most approaches assume to relax these requirements and model the abundances and variability of present taxa by fitting a distribution against real data. State of the art 16S count data simulators address the above simulation challenges using *ad hoc* statistical modeling such as Multinomial (McMurdie and Holmes, 2014), Dirichlet-Multinomial (Chen and Li, 2013), Gamma-Multivariate Hypergeometric (Patuzzi et al., 2019; Baruzzo et al., 2019) and large hierarchical models (Ma et al., 2021a). In this study, we assume that given realistic initial conditions, MiCRM addresses biological variability and produces realistic absolute abundances, while technical variability and noise are simulated with metaSPARSim (Patuzzi et al., 2019). We used metaSPARSim due to its modular nature, which allowed us to bypass the biological variability model and use only the sequencing step simulation. Furthermore, metaSPARSim offers complete control over sequencing simulation parameters and has been widely used in the evaluation of several 16S bioinformatics methods (Viljanen and Boshuizen, 2022; Khomich et al., 2021; Cappellato et al., 2022; Baruzzo et al., 2021) due to its performance in simulating realistic count data.

In summary, our work proposes a simulator that addresses a critical gap in the field of bacterial interaction network analysis and bridges known metabolic consumption networks with the data that is typically used for reverse engineering. This approach not only allows for the evaluation of algorithm performance across networks of varying connectivities, but also introduces a novel framework for simulating sequencing count data from MiCRM-generated networks. The primary goal of this work is to introduce N2SIMBA as a flexible and modular simulation framework capable of generating realistic synthetic 16S rDNA-seq count tables from any user-defined microbial interaction network with known structure, providing a principled gold standard for benchmarking network inference methods. As secondary goal we demonstrate its utility through two case studies: the first investigates how network topology and resource availability jointly shape bacterial community composition and persistence; the second provides a small-scale comparative evaluation of two widely used network inference methods applied to N2SIMBA-generated count data.

## 2 Materials and methods

To assess the effectiveness of reverse engineering methods, it is essential to have a gold standard, which means a network where the actual interactions driving the observed taxa abundances are known. Currently, there are no sufficiently large microbial networks with known interactions. Therefore, abundance data from synthetic sequencing with a known interaction network are required to compare the results of the methods. We introduce N2SIMBA, a simulation framework that includes i) the specification of the dynamics of the bacteria and the metabolites; ii) a clear definition of interactions between taxa in terms of network, e.g., an adjacency matrix; iii) a generative model for sequencing count data.

### 2.1 Simulation of bacteria and resources dynamics

To specify the dynamics of the system, we use Community Simulator (Marsland et al., 2020a), an open-source Python package that implements a MiCRM (Marsland et al., 2020b) and allows one to simulate the dynamics of microbial populations in a reproducible, transparent and scalable way. The model tracks the absolute abundance $N_{i}$ of each taxon (denoted i , where i ranges from one to S , with S representing the total number of taxa considered) and the concentration $R_{\alpha }$ of each metabolite α (denoted α , where α ranges from one to M , with M representing the total number of metabolites). The dynamics of the taxon abundances and metabolite concentration are governed by a system of ODEs described in detail in Section 1.1 of Additional file 1 as in (Marsland et al., 2019). Briefly, each taxon grows at a rate proportional to the energy surplus obtained from metabolite consumption after subtracting a minimum maintenance cost, while metabolite concentration change according to consumer uptake, metabolic by-product secretion, and intrinsic replenishment dynamics. The two main parameter matrices are the consumer preference matrix $C\in R^{S\times M}$ , describing the affinity $c_{i\alpha }$ of each taxon i for each metabolite α , and the metabolic matrix $D\in R^{M\times M}$ , describing the stoichiometric conversion rates $d_{\alpha \beta }$ between consumed α and produced β metabolites. To ensure energy conservation, the model additionally includes an environment node (ENV) and a waste metabolite w , not consumed by any taxon.

### 2.2 Define network of interaction between taxa

**FIGURE 1:**
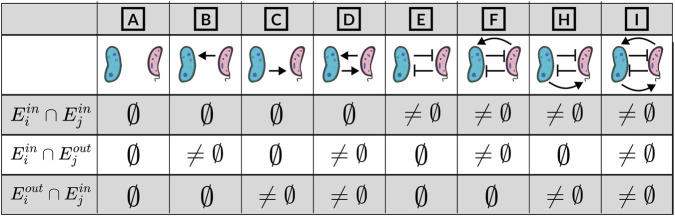
This figure reports all the possible interactions between taxa as intersections of the metabolites consumed and produced.

To understand the interactions between taxa mediated by metabolites, it is crucial to adopt a definition of the term interaction that takes into account the metabolites present in the environment. This involves examining the metabolites absorbed and released by different taxa, which form the basis for describing various types of interaction (Tschikantwa et al., 2018). Let $E_{i}^{in}$ denote the set of metabolites taken in as input by taxon i , and $E_{j}^{\mathrm{out}}$ represent the metabolites generated as output by taxon j . When considering two arbitrary taxa, i and j , all potential interactions can be elucidated by examining the intersection of metabolites consumed and produced, as illustrated in Figure 1. The MiCRM allows for the description of all these interactions, with the exception of toxicity. Toxicity is not allowed within the MiCRM framework since this framework does not account for the possibility that some metabolites may be toxic for a specific taxon i, rather than beeing an energy source. In the case of all other scenarios, it can generally be stated that positive interactions occur when one taxon consumes one or more metabolites produced by another taxon, while negative interactions arise when the two taxa share the consumption of at least one metabolite in common. For example, if two taxa do not compete for the same metabolite (i.e., $E_{i}^{in}⋂E_{j}^{in}=\emptyset$ ), and taxon j does not produce metabolites consumed by taxon i (i.e., $E_{i}^{in}⋂E_{j}^{\mathrm{out}}=\emptyset$ ), but taxon i produces metabolites consumed by taxon j (i.e., $E_{i}^{\mathrm{out}}⋂E_{j}^{in}\neq \emptyset$ ), then in the network there will be a positive edge from taxon i to taxon j . On the other hand, if two taxa have input metabolites in common $\left(E_{i}^{in}⋂E_{j}^{in}\neq \emptyset \right)$ , but none of the metabolic products are consumed by the two taxa (i.e., $E_{i}^{in}⋂E_{j}^{\mathrm{out}}=\emptyset \wedge E_{i}^{\mathrm{out}}⋂E_{j}^{in}=\emptyset$ ) then in the network there will be two negative edges between the two taxa.

It is worth noting that more intricate relationships can be delineated. For example, if two taxa consume the same metabolite, yet there exist metabolic by-products of taxon j that are consumed by taxon i , and not reciprocally (as depicted in Case F) Figure 1, the network will feature two negative connections between the taxa, along with a positive connection between taxon j and taxon i . Furthermore, it is crucial to emphasize that variations in the availability of metabolites at different time points can alter the nature and strength of interactions between taxa. For example, a high abundance of shared resources could significantly diminish negative interactions between taxa that compete for the same resources, whereas a scarcity of the same metabolite in the environment could lead to a pronounced negative interaction between the two taxa.

For the purpose of building a simulator to benchmark methods to reverse engineer bacterial interactions, we need to represent the network of bacterial interactions in the form of its adjacency matrix $A\in R^{S\times S}$ . In principle, it is always possible to build A from systems where the consumption matrices C and the metabolites D are known using the rules depicted in Figure 1 although, in some cases, we might not be able to univocally define the sign and direction of the interaction in the gold standard itself (Figure 1, Cases F, H and I).

Currently, as far as the authors knowledge goes, there exist no real bacterial interaction networks of large dimensions (i.e., from dozens to hundreds (Matchado et al., 2021)) where both the matrices C and D are known. To address this limitation, we have also developed an algorithm (i.e., module of *N2SIMBA*) capable of taking a directed network with specified topologies as input and generating the corresponding matrices C and D , as an alternative to starting from given C and D . In addition, this capability allows for a comprehensive assessment of the performance of the methods across networks that exhibit various types of topology.

*N2SIMBA* introduces some constraints. First, we model the absence of interaction, mutualism, commensalism and competition (Tschikantwa et al., 2018) considering the interactions depicted in Cases A), B), C), D) and E) of Figure 1 to avoid having edge signs that change over time depending on the availability of the metabolite. Second, we have chosen to define the matrices C and D in a way compatible with the network topology A that ensures one-to-one correspondence between the structures of A and that of C and D . Although it may not completely mirror biological reality, this guarantees the uniqueness of the network reconstruction task undertaken by the algorithm. This ensures that we evaluate solely the performance of the reverse engineering algorithm, rather than the unique *a priori* identifiability of the network.

**FIGURE 2:**
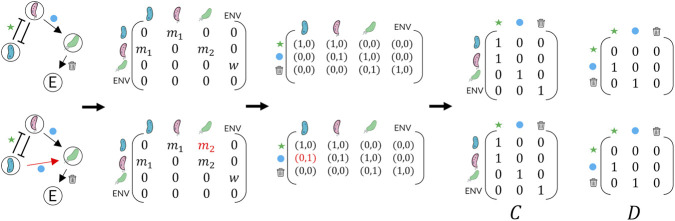
Ambiguity can arise in MiCRM simulations when two species compete to consume a shared resource. Consuming the latter allows both parts to produce a metabolite, therefore to have an outgoing edge to all other taxa that consume such resource. The figure denote how from two different networks, the same C and D matrices are interpreted.

To gain a clearer insight into this second aspect, it is crucial to acknowledge that, both in the Community Simulator and generally in MiCRMs, there is no functionality available to specify which taxa are responsible for the production of a specific metabolite. In the model, consumers are only aware of what they should consume and lack knowledge about which compatible producer taxa actually supply them. In theory, this information can be inferred from the metabolic matrix D . However, this would imply that all taxa that consume a specific metabolite would produce identical metabolite products. Not having the metabolic matrix specific for a taxon introduces an ambiguity, as illustrated in Figure 2, where two distinct interaction networks A could potentially correspond to the same C and D . Although this ambiguity represents an inherent limitation of MiCRM, considering the ultimate objective of establishing a gold standard for network inference methods, it is imperative to ensure that a given pair of C and D is unequivocally associated with one adjacency matrix A . To achieve this, additional interactions are introduced to the taxa involved in negative interactions, encompassing the union of their respective sets of positive outgoing edges. Specifically, we enforce that any outgoing edges, if present, from the two nodes engaged in a bi-directional negative interaction must be connected to the same node. For example, the network shown at the top of Figure 2 will be consistently transformed into the network at the bottom by our algorithm.

#### 2.2.1 Definition of consumer preferences and metabolic rules

*N2SIMBA* receives the adjacency matrix A as input and defines the matrices C and D , which represent an interpretation of the edges in A as metabolite-mediated interactions between pairs of taxa. A is either user-defined or generated using various state-of-the-art generation models, such as Erdős-Rényi (ER) random networks (Erdős and Rényi, 1958), Watts-Strogatz (WS) small world networks (Watts and Strogatz, 1998) or Barabási-Albert (BA) scale-free networks (Réka and Barabási, 2002) or Bollobas-Borgs (BB) (Bollobás et al., 2003). By Erdős-Rényi topology we refer to networks that have a comparably similar number of connections for each node and have a degree distribution that is better described by a Poisson distribution. Scale-free networks, instead, have been demonstrated to have a degree distribution that follows a power law (Réka and Barabási, 2002). For user convenience, we developed a set of functions to generate a wide range of network topologies based on the Python library NetworkX (Hagberg et al., 2008).

According to the definition of Community Simulator, consumer preferences are defined as a matrix $C\in R^{S\times M}$ where nonzero entries indicate which metabolites are consumed by each taxon. The set of metabolic rules, instead, is defined as a matrix $D\in R^{M\times M}$ , where the metabolites consumed and the ones produced are represented in the columns and in the rows, respectively. Nonzero entries in this matrix represent metabolic rules. It is important to bear in mind that, given the Community Simulator’s adherence to the energy conservation law, every node must have at least one incoming edge and one outgoing edge. Therefore, in matrix A , we must also include the node representing the environment (ENV). This node is then linked to all taxa that have either zero outgoing edges (with an edge from the taxon to ENV) or zero incoming edges (with an edge from ENV to the taxon). Similarly, M denotes the total count of available metabolites, which includes a waste metabolite *w*. This metabolite is not consumed by any taxon and is released into the environment.

The definition of consumer preferences and metabolic rules consists of three steps: i) assigning a metabolite to each edge in A ; ii) assigning the consumed and produced metabolites to each node; and iii) defining consumer preferences and metabolic rules.

##### 2.2.1.1 Metabolite-edge association

This algorithm (Algorithm 1) assigns a metabolite to each edge in A , representing the modulation of the interaction between the two taxa. The core principle is that positive edges sharing a target node are governed by the same metabolite, while each pair of competitive negative edges receives a distinct metabolite, preventing spurious co-regulations between otherwise unconnected nodes. A final waste metabolite w is assigned to all edges entering the ENV node. The algorithm takes A as input and return the total metabolite count M and the metabolite-edge matrix $M_{e}\in R^{S\times S}$ .

##### 2.2.1.2 Metabolite-nodes association

The metabolite-node algorithm (Algorithm 2) determines which metabolites each taxon consumes or produces, encoding this information in the three-dimensional array $M_{s}\in R^{M\times S\times 2}$ . The two levels of the third dimension distinguish consumption from production, where a positive edge signifies that the source node produces the metabolite consumed by the target, whereas a negative edge indicates that both nodes compete by consuming the same metabolite.

##### 2.2.1.3 Consumer preferences and metabolic rules

Algorithm 3 derives the consumer-preference matrix C and metabolic-rule matrix D directly from $M_{s}$ . Conceptually, C reflects which metabolites each taxon prefer to consume, while D encodes the conversion rates between consumed and produced metabolites. Both matrices are initially binary and can subsequently be scaled or sampled from appropriate distributions (Normal, Gamma, or Dirichlet) to introduce quantitative heterogeneity in consumption preferences and metabolic conversion rates. If scaled from binary, non-zero $d_{\beta \alpha }$ are normalized to the sum of columns of $d_{\alpha }$ to ensure that the sum of the columns is 1.

Algorithm 1 **Metabolite-edge association.**

**Input:** adjacency matrix **A**
**Output:** M; metabolite-edge matrix $M_{e}$

1: $M_{e}\leftarrow$ zeros matrix $\in R^{S\times S}$

2: m←0 ; j←1

3: **while** j<S **do** ⊳ While scanning all network nodes
4: $E_{j}^{+}\leftarrow$ set of nodes where $A_{\cdot ,j}>0$

5: $E_{j}^{-}\leftarrow$ set of nodes where $A_{\cdot ,j}<0$

6: **if** $E_{j}^{+}\neq \emptyset$ **then**⊳ If there are positive in-edges to node j

7: m←m+1

8: $M_{e}\left[E_{j}^{+},j\right]\leftarrow m$

9: **end if**
10: **if** $E_{j}^{-}\neq \emptyset$ **then**⊳ If there are negative in-edges to node j

11:   **for** $i\in E_{j}^{-}\wedge i>j$ **do**⊳ Iterate for each not seen yet in-edge
12: m←m+1

13: $M_{e}\left[i,j\right]\leftarrow m$ ; $M_{e}\left[j,i\right]\leftarrow m$

14:   **end for**
15:  **end if**
16: j←j+1

17: **end while**
18: $E_{S}^{+}\leftarrow$ set of nodes of $A_{\cdot ,S}>0$

19: M←w←m+1

20: **if** $E_{S}^{+}\neq 0$ **then**⊳ If there are positive in-edges in the ENV
21: $M_{e}\left[E_{S}^{+},S\right]\leftarrow w$

22: **end if**
**return** M; $M_{e}$

Algorithm 2 **Metabolite-nodes association.**

**Input:** M; adjacency matrix A ; metabolite-edge matrix $M_{e}$

**Output:** metabolite-species matrix $M_{s}$

1: $M_{s}\leftarrow$ zeros matrix $\in R^{M\times S\times 2}$

2: **for** i←1 to S **do** ⊳ For each node
3: $E_{i}^{\mathrm{out}}\leftarrow$ set of nodes where $A_{i,\cdot }\neq 0$

4: $E_{i}^{in}\leftarrow$ set of nodes where $A_{\cdot ,i}\neq 0$

5:  **for** $j\in E_{i}^{\mathrm{out}}⋃E_{i}^{in}$ **do**⊳ For each in- and out-edge of node i

6: $m^{\prime }\leftarrow M_{e}\left[i,j\right]$

7:   **if** A[i,j]>0 **then**⊳ If the edge (i,j) is positive
8: $M_{s}\left[m^{\prime },i,1\right]\leftarrow 1$ ; $M_{s}\left[m^{\prime },i,2\right]\leftarrow 0$

9: $M_{s}\left[m^{\prime },j,1\right]\leftarrow 0$ ; $M_{s}\left[m^{\prime },j,2\right]\leftarrow 1$

10:   **end if**
11:   **if** A[i,j]<0 **then**⊳ If the edge (i,j) is negative
12: $M_{s}\left[m^{\prime },i,1\right]\leftarrow 1$ ; $M_{s}\left[m^{\prime },i,2\right]\leftarrow 0$

13: $M_{s}\left[m^{\prime },j,1\right]\leftarrow 1$ ; $M_{s}\left[m^{\prime },j,2\right]\leftarrow 0$

14: **end if**
15: **end for**
16:**end for**
**return M;** $M_{e}$

Algorithm 3 **Consumer preferences and metabolic rules.**

**Input:** M; metabolite-species association matrix $M_{s}$

**Output:** consumer preferences **C**; metabolic rules **D**
1:**C** $\leftarrow M_{s}{\left[\cdot ,\cdot ,1\right]}^{T}$

2:**D** ← zeros matrix $\in R^{M\times M}$

3:**for** i←1 to S **do** ⊳ For each node
4: consumed metabolites ← set of α metabolite indexes where $M_{s}\left[\alpha ,i,1\right]=1$

5: produced metabolites ← set of β metabolite indexes where $M_{s}\left[\beta ,i,2\right]=1$

6: D[β,α]←1

7:**end for**
8: If required, sample rows of C according to chosen methodology
9: If required, sample columns of D according to chosen methodology
**return C; D**

### 2.3 Simulating absolute abundance and sequencing count data

Once the network topology and the dynamic equations governing the interactions between taxa and metabolites have been established, the next step involves simulating the abundance matrix. This matrix contains the abundance profiles of various biological samples, with each sample representing a different state of the same system (i.e., the same network derived from the matrices C and D ) resulting from an initial condition perturbation. We might imagine that biological samples are derived from different perturbations, different time points, or different conditions, depending on the experimental settings.

Different settings represent running different simulations to reach different steady states, where we denote $N^{ss}\in R^{S\times 1}$ the absolute abundances of the taxa once the MiCRM converges to the steady state from an initial condition. The perturbation of the steady states in different ways produces different biological samples of the same microbial community under different experimental conditions. We denote the perturbation of $N^{ss}$ by $N_{p}\in R^{S\times B}$ , where B is the number of biological samples. For example, each column of $N_{p}$ is related to $N^{ss}$ but differs by the environment, which is altered by increasing or decreasing the concentration of specific metabolites by a scale factor $p_{s}$ , we can generate different samples under different experimental conditions. However, each sample is derived using the same consumer preferences and metabolic rules and, therefore, the same bacteria interaction network. We denote the absolute abundances of the taxa after converging once again to steady state for each biological sample as $X\in R^{S\times B}$ .

The procedure for perturbing the environment offers the option to either automatically design perturbations or allow users to specifically define which resources are altered for each sample. The automatic design of perturbations varies depending on the choice of supply rate. When using a constant supply rate, $f_{p}$ perturbed resources are randomly chosen from the pool of non-zero supply rates, and their perturbation occurs through upscaling or downscaling of a factor $p_{s}$ the supply rates. Conversely, when the supply rate is turned off and resources are treated as a bolus introduced at the beginning of the simulation, the perturbation alters directly the concentration of the resources at steady-state that are nonzero. More details about the implementation are provided in Section 1.2 of Additional file 1.

To derive the final sequencing data, the sequencing process modeling developed in metaSPARSim can be leveraged. metaSPARSim uses a Gamma-Multivariate Hypergeometric model to simulate, respectively, the biological and technical variability characteristic of 16S sequencing count data. In our work, we used only the simulation of the technical variability of the count data due to the sequencing process, that is, the multivariate hypergeometric model (Equation 1), since the biological variability of taxa abundance across the B samples is generated through B resource perturbations. Let $Y\;\in N^{S\times B}$ the simulated count table and $Y_{k}$ the count vector $Y_{ik}$ for taxa i in sample k , then the technical variability is modeled using a Multivariate Hypergeometric distribution and parameterization as in (Johnson et al., 1997):

$$Y_{k}∼Mult.Hyper\left(n=l_{k},m=X_{k}\right)$$ (1)

where $l_{k}$ is the library size of sample k and $X_{k}$ is the vector of taxa abundances $X_{ik}$ for taxa i in sample k .

The sequencing depth parameters $l_{k}$ for each biological sample can be specified by the users, or taken from an available database of library sizes, learned from real data, available in metaSPARSim and further extended by (Cappellato et al., 2022).

metaSPARSim models mainly the bias related to the limited amount of material that can be sequenced in a single experimental run with parameterized sampling without replacement of $l_{k}$ draws for each sample k=1,…,B . This enforces the simulation of the count data to preserve compositionality, dependence between features and sparsity of the data. In particular, the relationships between the features that the multivariate hypergeometric model enforces skewness of the outcome, characteristic of real 16s rDNA-seq data.

### 2.4 Software

**FIGURE 3:**
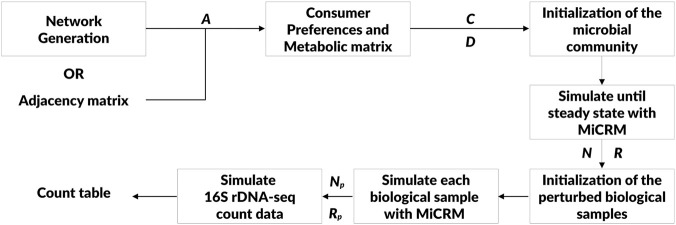
Scheme of the modular structure of N2SIMBA. Starting with either a provided or generated adjacency matrix, a bacterial interaction network is interpreted and a bacterial community is initialised and simulated with a MiCRM. The 16S rDNA-seq count data are then simulated and provided as output. *A* represents the adjacency matrix, *C* the consumer preferences matrix, *D* the metabolic matrix, *N* the bacterial abundances, *R* the resources in the environment, $N_{p}$ the bacteria abundances for each biological sample and $R_{p}$ the resources for each biological sample.

The simulation procedure, described in the Methods Section, is implemented in the Python package *N2SIMBA*. To enhance reproducibility and portability, the package is also available within a container in the same repository. The software has been tested and developed in a Linux environment using Python 3.10 (Van Rossum and Drake, 2009), and it is based on external libraries including NumPy (≥1.25.0) (Harris et al., 2020), pandas (1.5.3) (Mc Kinney, 2010), NetworkX (≥3.1) (Hagberg et al., 2008). An high-level scheme of the modular nature of *N2SIMBA* is presented in Figure 3. The implementation of the MiCRM relies on Community Simulator.

In cases where no adjacency matrix A is provided to the simulation procedure, users are given various options to generate networks using both the ER and BA models. The generated networks are directed and weighted, where the weights are all the same but have different signs. The positive edges are set to 1, and the negative edges are set to −1, where the ratio of negative edges, therefore competition between pairs of taxa, is parameterized with $n_{\mathrm{neg}}$ .

Although an ER network is already implemented in NetworkX, the package does not provide a directed network as an outcome of the BA model. To address this, we modify the preferential attachment process by randomly determining, with equal probability, how each new node added to the network will be connected to other nodes with an outgoing edge, an ingoing edge, or both (bidirectionally).

The package includes functions to generate the adjacency matrix A , to generate C and D , to initialize the microbial community and MiCRM, to run the free evolution to steady state, to initialize the perturbed steady state, and to run the simulation of the 16s RNA-sequence of the steady state taxa. The key parameters for initializing the microbial community and MiCRM are the model parameters and initial conditions. If the initial conditions are not known, the software requires the user to choose which preset will be used to automatically sample realistic initial conditions for the taxa abundances. The presets are provided from metaSPARSim (Patuzzi et al., 2019) and consist of 16s rDNA-seq sequenced data. The datasets are averaged between different replicates of the same experimental conditions.

The software includes a documentation section containing a notebook and straightforward usage tutorials, along with a methods section that delineates each step of the simulation procedure to improve the clarity and readability of the procedural workflow.

### 2.5 Case studies

To more rigorously illustrate the proposed simulation framework, we conducted two primary case studies involving BA and ER bacterial interaction networks. The objective of the first case study is to investigate how environmental resource availability and the underlying topology of bacterial interaction networks jointly influence the compositional structure and persistence of the bacterial community. The second case study is designed to provide a small-scale comparative evaluation of network inference methods based on count tables generated by N2SIMBA.

#### 2.5.1 Simulation of two bacteria interaction networks topologies

The networks were generated using the BA and ER models under varying conditions of resource availability, with parameters specified in Table 1. For each model, distinct network instances were produced by varying the number of species (S=50,100,150,250) and, respectively, the number of initial (seed) nodes (k=2,3,4) in the BA model and the connection probability between any pair of nodes (p=0.1,0.2,0.3) in the ER model. In the BA topology, seed nodes represent the initial nodes from which the scale-free network is generated and therefore tend to become highly connected hubs within the network (Réka and Barabási, 2002).

**TABLE 1:** Definition of the MiCRM parameters and the ones that are used to handle initialization of resource availability, perturbation of the biological samples and definition of the dynamic regime of all energy inputs within taxa. Additional parameters are reported in Supplementary Table S1 of the Additional File 1.

Symbol	Description	Value [unit of measurement]
$n_{\mathrm{neg}}$	Ratio of competitions	0.1 [unitless]
$m_{c}$	Unitary metabolic cost	1 [energy/time]
g	Conversion factor from energy uptake to growth rate	1 [1/energy]
l	Leakage factor	0.8 [unitless]
w	Energy content for resources	1 [energy/mass]
$R^{0}$	Intrinsic equilibrium abundance for resources	1000 [mass/volume]
$J^{in}$	Input energy flux to every taxon	Linear

These networks were subsequently employed to construct the matrices C and D using the procedure described in Section 3, which details the formal definitions of C and D . The matrix C was generated as a binary matrix, whereas the matrix D was normalized such that each column sums to one. Then, MiCRM was used to simulate the temporal dynamics of the bacterial community, requiring specification of initial resource concentrations and taxa abundances. Resource concentrations were controlled by the parameter vector $f_{r}$ (indicating the fraction of supplied resources), which was set to 0.05, 0.5, and one to represent, respectively, poor, moderately rich, and rich environments. The absolute resource levels were fixed to the value $R^{0}$ , as reported in Table 1. This modeling choice assumes that resources are homogeneous in the environment and supplied for a given time interval prior to taxa introduction.

Initial taxa abundances were parameterized to reflect empirical distributions observed in real 16S rDNA-seq count tables, using data from the metaSPARSim database. In particular, we employed the dataset denoted as *R1* by the original authors (Patuzzi et al., 2019).

The supply rate for all supplied resources was kept constant, and the internal energy influxes for each taxon were assumed to depend linearly on resource uptake. MiCRM simulations were run until convergence to a steady state. From each network, B=30 biological samples were generated. For each sample, both the fraction of perturbed resources $f_{p}$ and the magnitude of the perturbations $p_{s}$ were randomly drawn, with $f_{p}∼U\left(0,1\right)$ and $p_{s}∼U\left(2,10\right)$ .

The case studies are presented by generating B=30 biological samples, where for each perturbation a different number of resources was perturbed and scaled with a different strength.

Multiple networks were simulated by investigating both the network size (S) and the respective network parameters, the number of initial seed nodes (k) and the probability of connection between two nodes (p) , respectively, for the BA and ER topology. Then, those networks are interpreted as bacteria interaction networks using *N2SIMBA*, where we sampled C to remain binary and D to normalize to column sum. The initialization of the MiCRM requires the initialization of both the resource concentrations and the abundances of the taxa. The concentrations of resources are controlled by $f_{r}$ , which describes the percentage of the resources supplied and was fixed at the value of $R^{0}$ . This was supported by assuming that these resources are omogeneous in the environment and supplied for a while before introducing the taxa. We initialized taxa abundances to reflect the distribution observed in real 16S count tables, using data available in metaSPARSim’s database. Specifically, we selected the dataset identified as *R1* by the authors (Patuzzi et al., 2019). The dynamics of the MiCRM are presented in Table 1, where the supply rate is designed to remain constant for the supplied resources, and the input energy fluxes within the taxa are designed to be linear. Three different regimens of resource availability are designed to describe poor, moderately rich, and rich environments, respectively, with $f_{r}$ set to 0.05, 0.5, and 1. The MiCRM is allowed to evolve freely until it converges to a steady state, and B biological samples are generated by perturbing the supplied resources. Both the number of resources and the fold changes are randomly sampled, with $f_{p}∼U\left(0,1\right)$ for the number of resources and $p_{s}∼U\left(2,10\right)$ for the fold changes. Details about the perturbed environments are referenced in section 1.3 in Additional file 1.

#### 2.5.2 A small-scale comparison of network inference methods

The main purpose of the proposed simulation is to evaluate methods of microbial interaction inference. To this end, we present a small-scale benchmark to showcase the application of N2SIMBA and its validity as a simulator of microbial count data. We considered two methodologies commonly used to infer the interaction network of microbial communities: Pearson correlation and SPRING (Yoon et al., 2019). Pearson correlation serves as a ubiquitous, naive baseline; while it is a simple metric frequently used to measure microbial associations, it is notoriously susceptible to inferring spurious correlations due to the compositional nature of microbiome data (Friedman and Alm, 2012). By contrast, SPRING was chosen as a representative, state-of-the-art, microbiome-specific method. It relies on sparse partial correlations and explicitly models both the compositionality of the data and zero-inflation. For Pearson correlation, we used a modified $Y_{k}$ , for each k=1,…,B , with the $clr_{m}$ transformation to compute the association between taxa. Pearson correlation assumes that the data lie in an Euclidean space and the $clr_{m}$ transformation is applied to satisfy this assumption. The modified $clr_{m}$ is derived from the original one proposed by Aitchison (Aitchison, 1982) and is applied to the simulated 16S rDNA-seq table as follows:

$$clr_{m}\left(Y_{k}\right)=\left\{\frac{Y_{ik}}{g\left(Y_{k}\right)}\;|\;i\in I\right\}\cup \left\{Y_{ik}\;|\;i\notin I\right\}\;with\;I=\left\{i\in \left\{1,\ldots ,S\right\}\;|\;Y_{ik}\neq 0\right\}$$

In order to obtain the final interaction network, we determined statistically significant associations using an empirical permutation test defining a null hypothesis, to which the associations are tested. SPRING is based on the neighborhood selection method (Meinshausen and Bühlmann, 2006) and estimates the neighborhood of each node by solving a $\ell_{1}$ -regularized linear regression problem for each node, using a stability-based approach StAR (Liu et al., 2010) to select the regularization parameter. The result is a sparse precision matrix that under the assumption of multivariate normality can be interpreted as the conditional dependence graph of the bacterial species. SPRING adapts the neighborhood selection method to 16S data, first applying a variant of the clr transformation and then computing a semi-parametric rank-based covariance matrix, in place of the Pearson sample covariance matrix.

We ran N2SIMBA using both ER and BA models to evaluate the performance of the inference methods. For each model, we used two values for the network generation parameters p∈[0.05,0.15] and k∈[2,6] , respectively. For each topology and network generation parameter, we considered two sample sizes B∈[100,300] for a total of eight different scenarios. The other simulation parameters were kept fixed at $S=50,\;n_{\mathrm{neg}}=0.05,\;f_{p}=0.1$ and a Type III dynamic response input flux (Hill function) with $\sigma_{\max}=5$ , $n_{\mathrm{hill}}=2$ . More details on simulation parameters are found in Supplementary Table 1. For each parameter configuration, we simulated 50 iterations to obtain statistically robust results, for a total of 400 simulated datasets.

The tested inference methods consider cross-sectional datasets and thus can only infer undirected networks, therefore, we converted the ground truth network from a directed to an undirected network. We assessed the performance of each method in two main ways. First, we measured their ability to correctly infer edges using the Matthews correlation coefficient (MCC)

$$MCC=\frac{TP\times TN-FP\times FN}{\sqrt{\left(TP+FP\right)\left(TP+FN\right)\left(TN+FP\right)\left(TN+FN\right)}}$$

MCC measures the efficacy of the methods in identifying the edges (we can think of the inference procedure as a classification of all node pairs with two labels one if the edge is present and 0 otherwise) and ranges between −1 and 1, where MCC=0 represents the performance of a random classifier.

Second, we compared the inferred networks with the ground truth ones in terms of topological and structural properties, focusing specifically on network diameter D , radius R , average distance $\bar{\delta }$ , average clustering coefficient $\bar{C}$ , degree distribution and betweenness centrality distribution B(z) .

$$\begin{array}{ll} D & =\underset{i\in V}{\max}\left(\underset{j}{\max}\delta_{ij}\right)\;R=\underset{i\in V}{\min}\left(\underset{j}{\max}\delta_{ij}\right)\\ \bar{C} & =\underset{i\in V}{\sum }\frac{C\left(i\right)}{S}\;\;\bar{\delta }=\underset{i,j\in V}{\sum }\frac{\delta_{ij}}{S\left(S-1\right)}\\ & \;\;B\left(z\right)=\underset{i,j\in V}{\sum }\frac{\sigma \left(i,j|z\right)}{\sigma \left(i,j\right)} \end{array}$$

with $\delta_{ij}$ being the distance between two nodes, defined as the length of the shortest path that connects the two nodes; $C\left(i\right)=\frac{2e_{i}}{deg\left(i\right)\left(deg\left(i\right)-1\right)}$ being the clustering coefficient of node i , where deg(i) is the degree of the node and $e_{i}$ the edges between the neighbors of i ; σ(i,j) being the number of shortest paths between two nodes and σ(i,j|z) the number of shortest paths between the two nodes that pass through node z . The diameter and radius are undefined when the graph is not connected. In these cases, we defined the diameter as the maximum diameter of the connected components and the radius as the minimum radius of the connected components (in the cases where the smallest component was an isolated node, we considered the radius as undefined).

## 3 Results

The results are organized into two case studies, described in Section 2.5, to assess the applicability of N2SIMBA in terms of both reliability and usability for network inference methods.

The first case study focuses on evaluating the reliability of *N2SIMBA* by analyzing whether the algorithm maintains the characteristics of the generated networks with BA and ER models after deriving the C and D matrices. It also investigates how taxa abundance distributions change with varying simulation parameters and assesses scalability by measuring execution time and memory usage as $n_{\mathrm{neg}}$ , S and the topological parameters k and p are varied.

The dimensions of the network are tested by changing the amount of taxa S between 50, 100, 150, and 250 species, while the topological parameter k describes the number of initial seed nodes for the scale-free topology and p be the probability of having a pair of taxa connected between each other for the ER topology. Further analyses show the effect of resource availability on the behavior of the MiCRM by changing the resource regime between poor, moderately rich, and rich described in Methods’s Section and how the perturbations of the achieved steady states are capable of generating different biological samples.

The second case study showcases the application of two methods of inference of microbial interaction networks applied on the output of N2SIMBA with varying parameters of network generation. Further analyzes elaborate on both the performance of the network in reconstructing the bacteria interaction network in input and the structural properties of the inferred networks, compared to the one in the input, assumed as ground truth.

All simulations are performed on a single core of a computing node equipped with 8 Intel(R) Xeon(R) Platinum 8260 CPUs. Runtime and RAM usages are monitored using the Slurm built-in analytics.

### 3.1 Case study: simulation of two bacteria interaction networks topologies

#### 3.1.1 Scale-free bacteria interaction network

The first set of analyzes explored scale-free bacteria interaction networks generated using the Barabási–Albert (BA) generation model. The networks were generated by varying the number of nodes S and the initial seed nodes k , representing the hub nodes of the main networks of bacteria interaction. The parameter k ranged from two to 4. Our investigation revealed that the bacteria interaction networks maintained key topological characteristics, such as in- and out-degree distributions and clustering coefficients, consistently between different values of S and k . Further details can be found in Subsection 2.1 of the Additional file 1. This consistency demonstrates that *N2SIMBA* is robust in preserving a scale-free topology as both the number of taxa and the number of initial seed nodes increase.

**FIGURE 4:**
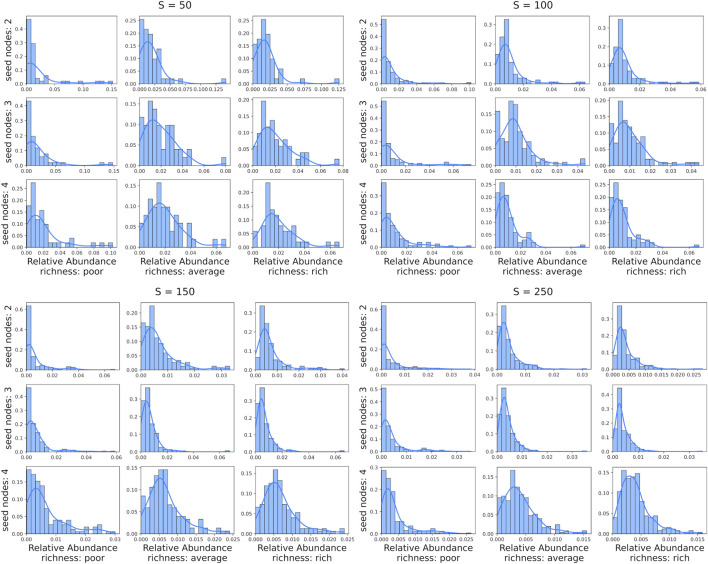
The taxa relative abundance distributions, with respective kernel density estimation, at steady states are reported in a multi-panel, where each panel is related to a different number of taxa S . From upper left to lower right we report S equal to 50, 100, 150 and 250. Then, each panel reports a grid of nine subplots, where the columns are related to the resource availability (from left to right, poor, moderately rich and rich) and the rows are related to the number of initial seed nodes (from top to bottom, 2, three and 4).

After interpreting bacteria interaction networks with the set of rules C and D , MiCRM is initialized by varying the resource availability regimen and freely evolves until it converges to steady state. What stands out from the steady-state taxa relative abundance distribution analysis reported in Figure 4 is that, with increasing resource availability, the distribution slightly shifts to the right by maintaining a right-skewed power law distribution. It appears that the majority of the species are able to survive and have a non-zero portion of relative abundance when the resource availability increases. A poor environment does not provide enough resources to maintain the survival of all nodes, and the larger the network, the more pronounced this behavior is. Weaker bacterial species are expected to struggle more to survive in a poor environment, such as, for example, unfertilized soil, characterized by low resource availability, compared to a rich environment, such as the gut, with abundant resource availability.

Furthermore, an increase in the number of initial seed nodes shows a reduction in the relative abundance gap between seed nodes and nodes in their neighborhood of radius one to 2. Consequently, this leads to a shift of the power law distributions towards a right-skewed bell-shaped distribution or a distribution resembling a log-normal distribution. This reduces the influence of the seed nodes on the relative abundance of the denser segment of the network and increases the contribution of the remaining nodes.

**FIGURE 5:**
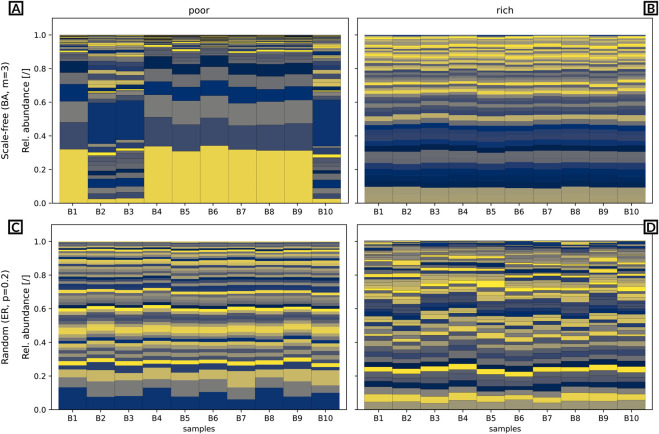
Taxonomic composition of ten samples from the simulated count data. The top panels **(A)** and **(B)** report the results for the BA network while the bottom panels **(C,D)** for the ER network. Columns are related to increasing resource availability from left to right (poor and rich). Within a panel, the colors represent the same species. For each panel the species are displayed by sorting them using their average rank between samples.

The different samples were generated by perturbing the steady-state environment, therefore changing the supply of resources as described in Methods’s Section. As an example, Figure 5 panels A and B show the taxonomic composition of ten samples from the simulated count data generated from the following bacteria interaction network settings: S=50 , k=3 , and $n_{\mathrm{neg}}=0.10$ under the conditions of poor and rich resource availability. The simulations reveal that the magnitude of compositional shifts driven by resource perturbations is strongly dependent on the baseline level of resource availability for a network of fixed size. Under poor resource conditions (Figure 5, Panel A), perturbations have a large effect on community composition: since fewer resources are available in total, even modest perturbations alter the supply of substantial fraction of what sustains the community, causing some species failing to survive entirely and others dominating as a result, showing a *rich gets richer* effect. As resource availability increases (Figure 5, Panel B), the same perturbations represent a smaller effect and the community composition stabilises with less pronounced variation across samples. The taxa that consistently thrive across samples are those connected to the ones with the largest degree in the bacteria interaction networks, and this effect is amplified in larger networks.

**FIGURE 6:**
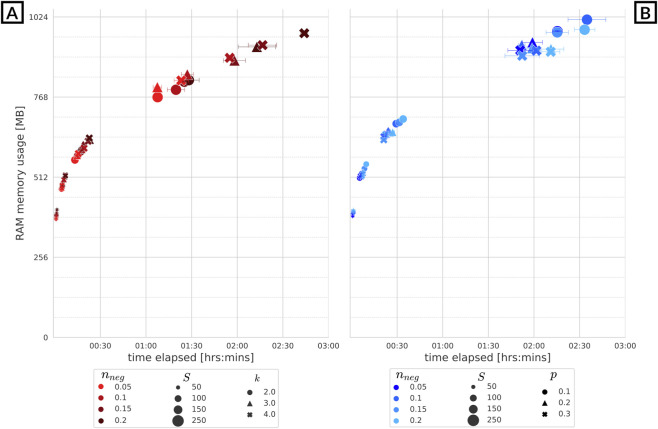
Computational burden in terms of runtime (x-axis) and peak memory usage (y-axis) averaged on three simulation runs (sd are represented as error bars). Computational burden is evaluated for each experimental settings by simulating B=100 biological samples varying the ratio of competition $\left(n_{\mathrm{neg}}\right)$ , the number of species (S) , the probability of connection between two nodes (p) in a ER network and the number of initial seed nodes (k) in a BA network. BA’s network metrics are reported on left panel **(A)**, while ER’s network metrics on right panel **(B)**. Simulation parameters $n_{\mathrm{neg}}$ , S , p and k are encoded using different colors, marker sizes and marker shapes, respectively.

In Panel A of Figure 6 the computational burden of different simulations of B=100 samples is shown when changing the experimental settings of a bacteria interaction network with a scale-free topology. The purpose of Figure 6 is to investigate the impact of the experimental settings while the energy input fluxes within the taxa are set to be linear. The results show that the size and number of initial seed nodes significantly impact computational times, while the ratio of competitive interactions has a minor impact on performance. Overall, *N2SIMBA* shows computational times in the range of hours for the harder experimental settings and RAM memory usage of around one Gigabyte at most.

#### 3.1.2 ER bacteria interaction network

Taking into account bacterial interaction networks that follow a random topology, generated using the ER model, each pair of taxa may have an interaction with probability p . This probability reflects the density of the bacteria interaction network and, in these analyzes, ranges between values of 0.1, 0.2, and 0.3.

**FIGURE 7:**
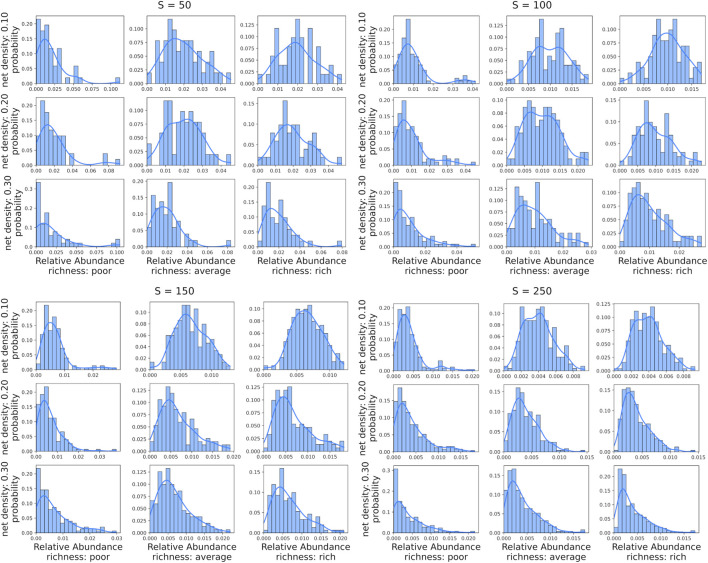
The relative abundance distributions of the steady states of the taxa of microbial communities driven by a ER’s network. The distributions are reported in a multi-panel, where each panel is related to a different number of taxa S . From upper left to lower right we report S equal to 50, 100, 150, and 250. Then, each panel reports a grid of nine subplots, where the columns are related to the resource availability (from left to right, poor, moderately rich and rich) and the rows are related the probability of interaction between each pair of taxa (from top to bottom, 0.1, 0.2 and 0.3).

Once the bacteria interaction network is defined, analyses are performed to investigate the effect of the network topology on the steady-state abundance of the taxa involved in the community. Figure 7 shows the steady state of the MiCRM initialized following the procedure explained in Methods’s Section with different characteristics of the bacteria interaction network and different resources availability. Increasing resource availability allows more taxa to receive many sources of energy and shifts the MiCRM to converge to a steady state where the relative abundances of the taxa follow a bell shape, better described from a Poisson distribution. This behavior is more evident when the network is sparser; we assume that this is due to the lower amount of competitions that depend on the number of edges that are present in the network. In fact, Figure 7 reports the results of bacteria interaction networks with $n_{\mathrm{neg}}=0.10$ , however, increasing $n_{\mathrm{neg}}$ shows that microbial communities with the same characteristics of the network (i.e., size and probability of interaction) converge to a steady state where the distribution of the relative abundances of taxa is harder to follow a bell shape, but remains closer to a right-skewed power-law distribution. More details are reported in Section 2.2 of the Additional file 1.

Panel C and D of Figure 5 reports the taxonomic composition of ten samples from the simulated count data generated from the experimental setting with the ER’s topology of taxa S=50 , p=0.2 and $n_{\mathrm{neg}}=0.10$ , across poor and rich resource availability conditions. In contrast to the BA case, the ER network produces a markedly more Poisson distribution of interactions across taxa and this structural difference is reflected in the community composition. When the environment is poor the ER network shows a higher robustness to environmental perturbations than the BA network, and this effect remains consistent when the richness of the environment increases. The residual inter-sample variability that does arise reflects the stochastic nature of the perturbations, which randomly benefit some taxa while imposing harder conditions on weaker ones.

Panel B of Figure 6 shows the comparison with the computational burden of simulating a microbial community driven by a network of bacteria interaction with a scale-free topology. There are no significant differences between different topologies; however, we note that a ER’s bacteria interaction network increases in computational burden the sparser it is. Furthermore, increasing the ratio of competitive interactions increases the complexity, resulting in greater participation of unique resources and an increase in the dimensionality of the MiCRM and the number of joint ODEs to compute.

### 3.2 Case study: a small-scale comparison of network inference methods

**FIGURE 8:**
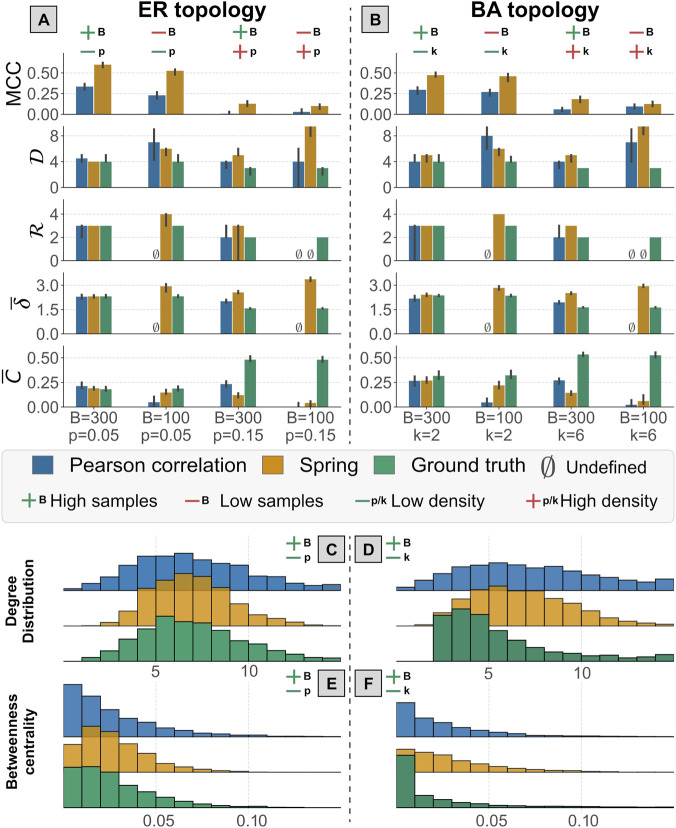
Results of the small-scale benchmark about Pearson correlation and SPRING performance in inferring the interaction network on data simulated using N2SIMBA. The top two panels **(A,B)** report the median values (with error bars representing the 25 and 75 percentiles) of MCC , diameter (D) , radius (R) , average distance $\left(\bar{\delta }\right)$ and average clustering coefficient $\left(\bar{C}\right)$ in the ER topology **(A)** and in the BA topology **(B)**. Each tick along the x-axis represents a different combination of simulation parameters B∈[100,300] and p∈[0.05,0.15], k∈[2,6] . The bottom panels show the network distributions for the scenario with B=300 and p=0.05, k=2 (for the other scenarios see Supplementary Figures S19, 20). Specifically, the degree distribution for the ER model **(C)** and BA model **(D)** and the betweenness centrality for the ER model **(E)** and BA model **(F)**. In each panel the true distribution (green) is compared against the distributions obtained using Pearson correlation (blue) and SPRING (yellow). The red and green plus/minus signs are visual indicators of the simulation parameters used. The green plus sign corresponds to B=300 , the red minus sign to B=100 , the red plus sign corresponds to p=0.15, k=6 and the green minus sign to p=0.05, k=2 . The symbol ∅ is used when the metrics are undefined. For example, the average distance is undefined when the network is not fully connected and the radius is undefined when there are isolated nodes with no connections.

The ability of N2SIMBA to simulate a realistic 16S count table from a known interaction network enables the evaluation of bacterial interaction inference methods. These methods take a 16S count table as input and output a network of tentative species interactions. Here, we tested the performance of two popular methodologies used to infer microbial interactions: the Pearson correlation method and the SPRING method based on partial-correlation. We simulated two sample sizes B∈[100,300] and two network generation parameters, p∈[0.05,0,15] for the ER model and k∈[2,6] for the BA model. It is important to note that increasing levels of the generation parameters lead to denser true interaction networks. We observed in Figure 8, Supplementary Figures S19, 20 that for a high sample number (B=300) and low density ( p=0.05 or k=2 ) both the methods had the highest performance for both the ER and BA topology. Figure 8 shows two groups of metrics to compare SPRING and Pearson accuracy in reconstructing of the ground truth: with the MCC and high-level topological descriptors of the networks (Panels A and B) and the node-wise centrality distributions (Panels C, D, E and F). SPRING outperforms Pearson in the reconstruction of the ground truth in terms of MCC , while it is comparable in reconstructing the topological descriptors. The reconstruction of the node-wise centrality distribution shows that SPRING recovers well the degree distribution in the ER topology under the case of high number of samples and low network density, while Pearson correlation infers a flatter distribution, as reported in Panel C of Figure 8. In the BA model, both methods struggled to infer the correct degree distribution (Figure 8D), suggesting that scale-free topologies are harder to infer, as observed in other studies (Kurtz et al., 2015; Hirano and Takemoto, 2019). In terms of betweenness centrality, a measure of node importance in the graph, we observed an acceptable qualitative overlap of the inferred distribution with the one of the ground truth, even if no method was able to obtain a perfect match, as reported in Panels E–F of Figure 8.

Finally, we can observe how the performance of the methods rapidly degrades as the number of simulated samples decreases or the density of the true network increases (Panels A–B of Figure 8). The fact that the number of samples has a considerable impact on the inference is not surprising, but considering the steepness of the decrease and the fact that in metagenomics studies the number of samples is generally low this highlights the need for careful consideration. Furthermore, it is quite evident that network density has a strong effect on the performance of the methods’, in agreement with previous studies (Hirano and Takemoto, 2019). This case study, although not intended as a comprehensive benchmark, highlights the importance of data simulation for understanding the applicability of bioinformatics methods.

## 4 Discussion

In this work, we proposed a bacterial network simulator, N2SIMBA, useful for benchmarking methods of reconstructing bacterial networks from metataxonomic data. The proposed procedure aims to simulate *in silico* count tables of a microbial community modeled with a known bacteria interaction network. The method is modular and consists of: i) simulating the interaction topology between bacteria, i.e., the adjacency matrix A ; ii) constructing the consumption matrix C and the metabolite matrix D from A using the *N2SIMBA* algorithm; iii) simulating the dynamics of bacteria and metabolites using an MiCRM framework; iv) simulating experimental perturbations leading to the generation of absolute abundances; v) simulating 16S sequencing of the samples. A scheme of N2SIMBA design is reported in Figure 3.

Results show that the *N2SIMBA* algorithm maintains, after deriving the C and D matrices, the characteristics of the BA’s and ER’s generated networks and that the count data show realistic distributions. In particular, when studying the distribution of species abundances as the parameters of the networks vary, we noticed that counts are distributed in a manner similar to the topology of the network used to model the microbial community. However, further analysis is needed to confirm this observation and understand its theoretical properties.

In the literature, there is no clear consensus on which network model better describes microbial interactions, although the scale-free model is often proposed (Faust and Raes, 2012b; Steele et al., 2011; Chaffron et al., 2010; Zhou et al., 2010). Therefore, a simulation framework should be flexible enough to simulate a bacteria community and the corresponding 16S count table, given any underlying interaction network. Remarkably, N2SIMBA takes as input an adjacency matrix, therefore, it can simulate any microbial interactions network, whose topology is provided as input.

One of the main limitations of N2SIMBA is that, being based on MiCRM, it implicitly assumes a single species-independent chemical reaction for the production of metabolites. However, in reality, the synthesis of a specific metabolite typically depends on the consumption of other metabolites, and such dependencies can vary between bacterial species. Modeling species-independent chemical reactions would require to consider a taxon specific D matrix, enabling a unique and not ambiguous pair of A and ( C ; D ) matrices without the requirement to introduce additional interactions described in Section 2.2. In future work, as more comprehensive data on species-specific metabolic pathways become available, more realistic simulation frameworks could replace the matrix D with explicit taxon-specific biochemical reactions, allowing to define more general interaction matrices A .

A related and broader limitation concerns the fact that not all microbial interactions are mediated by metabolite exchange. Microbial communities rely on a wider repertoire of interaction mechanisms including quorum sensing, contact-dependent inhibitions and asymmetric inhibitory interactions such as toxicity (Ghoul and Mitri, 2016). As a consequence of using MiCRM, N2SIMBA reflects only on a subset of the ecological relationships shaping real microbial communities and future extensions will explore the integration of communication-based interactions alongside the metabolic-based ones, for instance by incorporating quorum-sensing module that modulate growth rates as a function of local cell densities and define bacteria coordinate collective behaviors.

An additional potential future development, not currently implemented due to substantial modifications required in the overall N2SIMBA framework, involves categorizing M metabolites and S taxa into specific classes and families. In practice, metabolites can be categorized into distinct classes, such as sugars, lipids, and acids, each encompassing a specific subset of metabolites. In contrast, taxa can be grouped into various families, representing organisms that exhibit a preference for metabolites belonging to a particular class. In addition, it may be pertinent to consider the inclusion of a generalist family, which does not exhibit a specific preference for any particular class of metabolites.

Finally, our simulation of 16S rDNA sequencing is subject to several well-known biases, including gene copy number variation and amplification bias (partially due to primer mismatches). These biases were not modeled in the metaSPARSim rRNA-seq count simulator. Thanks to the modular nature of N2SIMBA, however, future simulation tools that explicitly model these biases could be readily integrated as substitutes for metaSPARSim, allowing for more realistic and bias-aware synthetic datasets.

To the best of our knowledge, within the context of benchmarking metataxonomic data, this work is the first to consider relationships between bacteria that can interpret their behavior and be mediated by metabolites. We are aware that interactions between bacteria, if modeled with a graph, are represented with a single edge and mediated by a single metabolite, losing realism. There are other approaches to model the evolution of microbial communities, as highlighted in the reviews by (Srinivasan et al., 2024; Cappellato et al., 2021; Pasqualini et al., 2023). However, none of them use a known network topology as input and therefore do not allow the design and control of it as a simulation parameter. Since N2SIMBA is designed in a modular way, alternatives to each module may be used and integrated. For instance, the community simulation step could be replaced by other consumer-resource model implementations such as MiaSim (Gao et al., 2023), and the sequencing simulation could be substituted with simulators of alternative sequencing techniques, such as shotgun metagenomics, provided compatibility with N2SIMBA’s count table output is maintained.

## Data Availability

The original contributions presented in the study are included in the article/Supplementary Material, further inquiries can be directed to the corresponding author.
