# Supplementary material for: N2SIMBA: from Network topology to SIMulation of interactions and BActerial abundance, using microbial consumer resource model
Source: Front Bioinform. 2026 May 20;6:1783447. doi: 10.3389/fbinf.2026.1783447 (PMC13230013; doi:10.3389/fbinf.2026.1783447)
Supplement: Supplementary file 1 [file DataSheet1.pdf]

Supplementary Material

1 SUPPLEMENTARY METHODS

1.1 Microbial Consumer Resource Model (MiCRM)

The Microbial Consumer Resource Model (MiCRM) is a state-space model based on Ordinary Differential Equations (ODEs) that describes the dynamics of a microbial community in terms of taxa abundances and resource concentrations (Marsland et al., 2020b). The model focuses on the interplay between consumers (i.e., bacteria), which utilize and produce resources, and the resources themselves, which represent substances such as nutrients or other energy sources. The abundance of taxa and the concentration of resources constitute the entire state of the microbial community at any given time (Marsland et al., 2019).

The model describes the dynamics of taxa in terms of energy flows mediated by the consumption, secretion, and chemical transformation of M metabolites. Each taxon i receives energy through the consumption of metabolites, resulting in an energy flow J_i^{in} entering the taxon. A portion J_i^{out} of this energy is released into the environment as metabolic by-products, while the remaining energy J_i^{growth} is used for cell growth and replication. This adheres to the principle of energy conservation:

$$J_i^{in} = J_i^{growth} + J_i^{out} \tag{S1}$$

The dynamics of taxa abundance and metabolite concentration are governed by the following system of ODEs:

$$\frac{dN_i}{dt} = g_i N_i \left(J_i^{growth} - m_{c_i} \right) \tag{S2}$$

$$\frac{dR_\alpha}{dt} = h_\alpha(R_\alpha) - \sum_i^S \frac{1}{w_\alpha} N_i J^{in}(c_{i\alpha}, R_\alpha) + \sum_i^S \sum_{\substack{\forall \beta \\ \beta \neq \alpha}} \frac{1}{w_\alpha} N_i J^{out}(d_{\alpha\beta}, c_{i\beta}, R_\beta) \tag{S3}$$

where N_i denotes the absolute abundance of taxon i ($i = 1, \dots, S$) and R_α the concentration of metabolite α ($\alpha = 1, \dots, M$). Each individual of taxon i requires a minimum energy m_{c_i} to sustain its metabolic cost, and grows at a rate proportional to the remaining energy with coefficient g_i. The metabolite dynamics depend on input and output mass flows, obtained by dividing energy flows by the energy density of the metabolite w_α. The term $h_\alpha(R_\alpha)$ describes the intrinsic dynamics of metabolite α in the absence of consumers, which can be configured as: absence of replenishment, constant replenishment from outside, or self-regeneration.

A taxon i absorbs metabolite α in proportion to both its concentration R_α and the consumer preference $c_{i\alpha}$. For each metabolite β consumed, a metabolite α is produced with a conversion rate $d_{\alpha\beta}$.

The two main parameter matrices of the MiCRM are the consumer preference matrix $C \in \mathbb{R}^{S\times M}$, where each entry $c_{i\alpha}$ describes the preference of taxon i for metabolite α, and the metabolic matrix $D \in \mathbb{R}^{M\times M}$, where each entry $d_{\beta\alpha}$ describes the stoichiometric coefficient of metabolite β produced from the consumption of metabolite α. To ensure adherence to energy conservation, the model additionally includes an environment node (ENV) and a waste metabolite w, which is not consumed by any taxon and is released into the environment by all taxa.

The MiCRM is implemented in N2SIMBA via Community Simulator (Marsland et al., 2020a), an open-source Python package that provides a reproducible, transparent, and scalable framework for simulating complex microbial communities under various experimental scenarios.

1.2 Initialization of microbial communities

In this section we detail the procedure to initialize the initial conditions of the MiCRM of a microbial community. The initial abundances of taxa are known or are automatically derived from the presets in Patuzzi et al. S taxa are sampled from non-zero intensities of the presets, where intensities represent the mean value of 16s rDNA-seq count data of multiple replicates. Therefore, the S taxa are sampled without replacement from a realistic distribution of sequencing count data that does not consider the sparsity. The initial concentrations of resources depend on the supply regime chosen in the design of the experiment. If there is no supply of resources, the latter are initialized as a bolus of concentration at time $t = 0$ of value R^0. While, if the supply is given from outside the microbial community, the resource concentration is initialized at the value of the supply rates, however, there is a continuous supply for the whole evolution of the microbial community. The amount of resources that are initialized, in both cases depend, on f_s, which controls how many of the available resources are actually initialized at $t = 0$.

1.3 Initialization of perturbed environments

In this section, we detail the procedure to perturb the steady state of the microbial community. The perturbations change the environment by altering the resource concentrations. The changes depend on the type of supply that is designed in the experimental settings. If there is no supply of resources, but they are provided as bolus, then, the perturbations alter directly the resource concentration. While, if the supply is external and omogeneous, then the perturbations alter the value of the supply rate that is provided to the environment. Each different sample consists of a different set of resources that are chosen to be perturbed. f_r control the amount of available resources that are perturbed for each sample. For each available resource that is chosen to be perturbed, there is equal probability of being scaled up or down by a strength value p_s that is designed or extracted from a uniform distribution of the designed interval.

2 FIGURES

2.1 Bacteria interaction networks characteristics

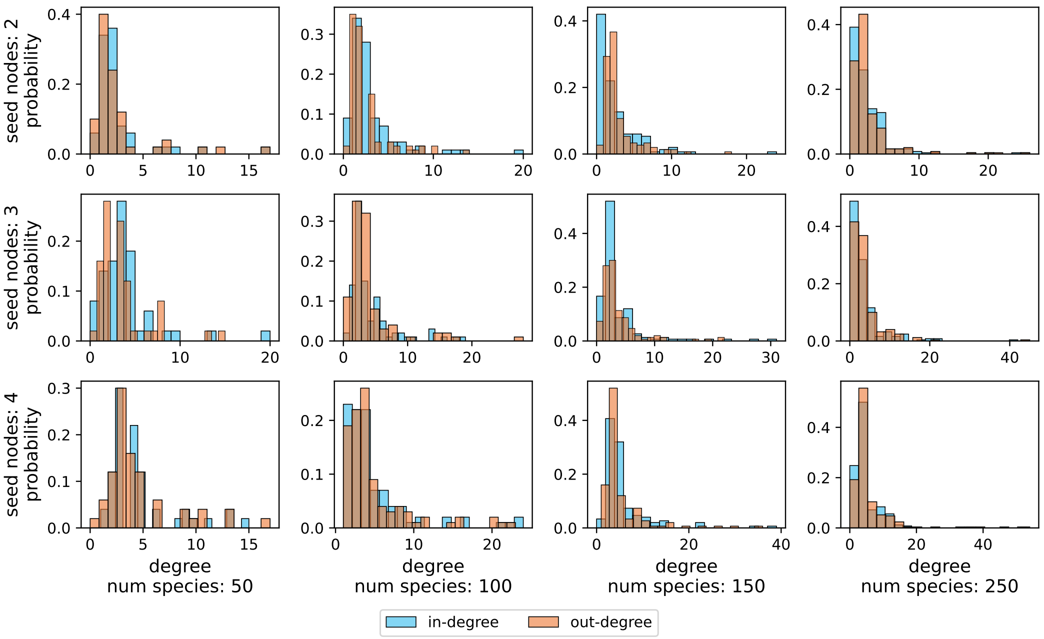

Figure S1: The figure reports the in- and out-degree distribution of the preprocessed bacteria interaction network with scalefree topology and $n_{neg} = 0.00$

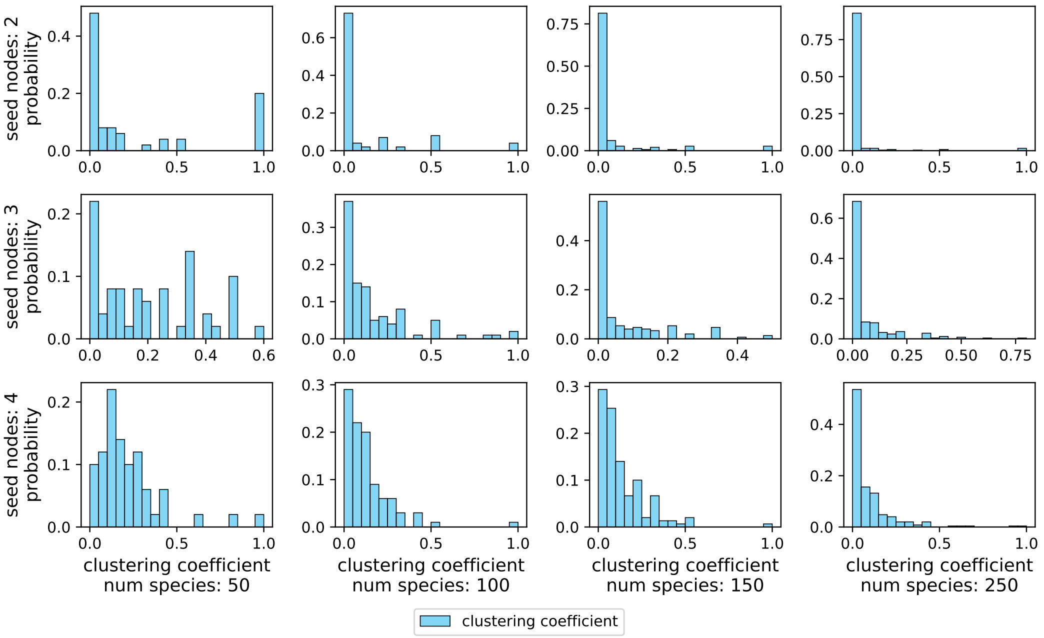

Figure S2: The figure reports the clustering coefficient distribution of the preprocessed bacteria interaction network with scalefree topology and $n_{neg} = 0.01$

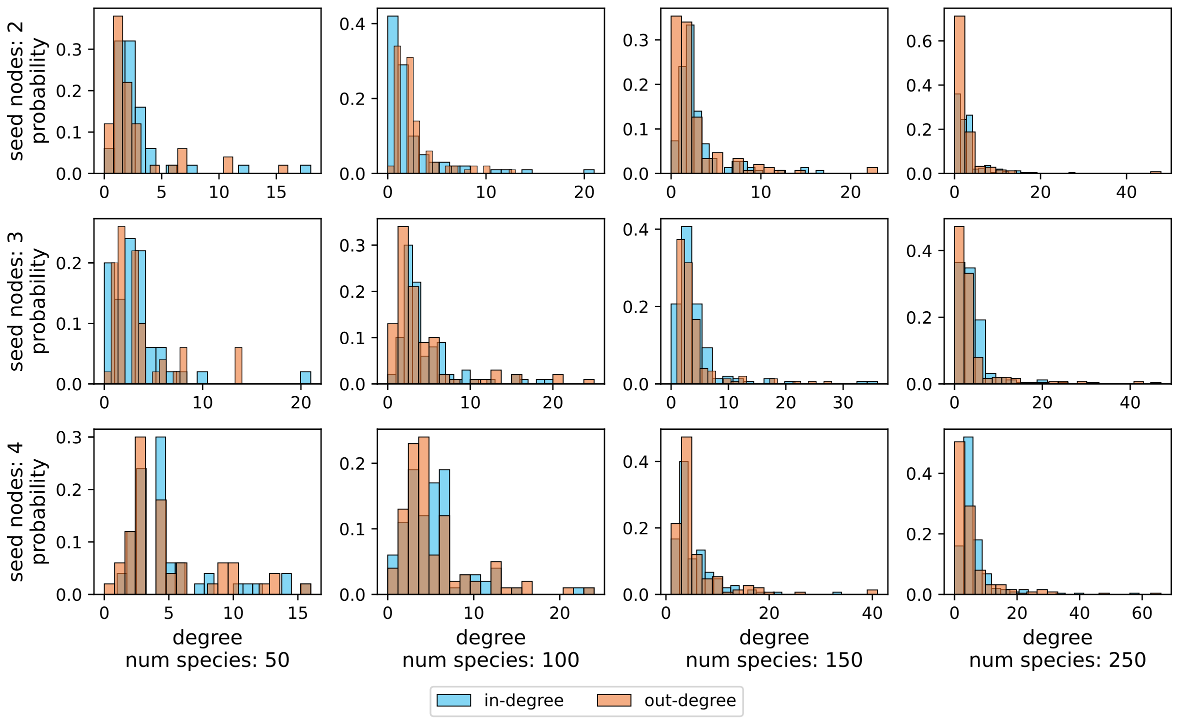

Figure S3: The figure reports the in- and out-degree distribution of the preprocessed bacteria interaction network with scalefree topology and $n_{neg} = 0.01$

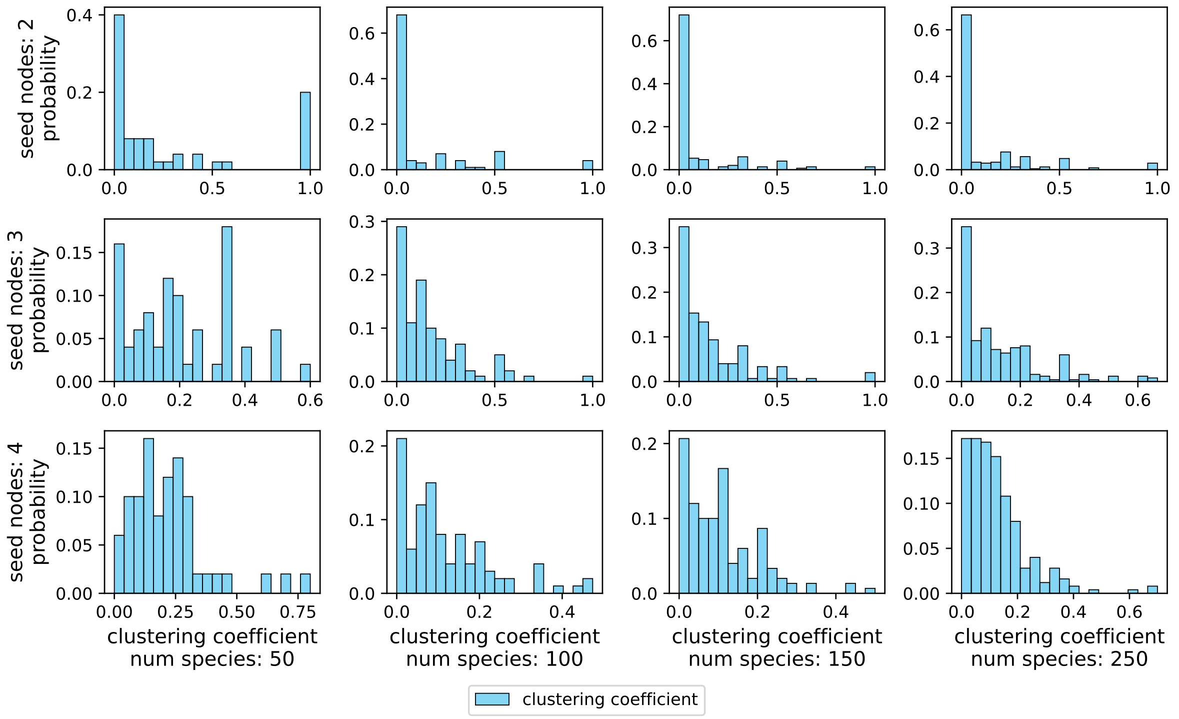

Figure S4: The figure reports the clustering coefficient distribution of the preprocessed bacteria interaction network with scalefree topology and $n_{neg} = 0.01$

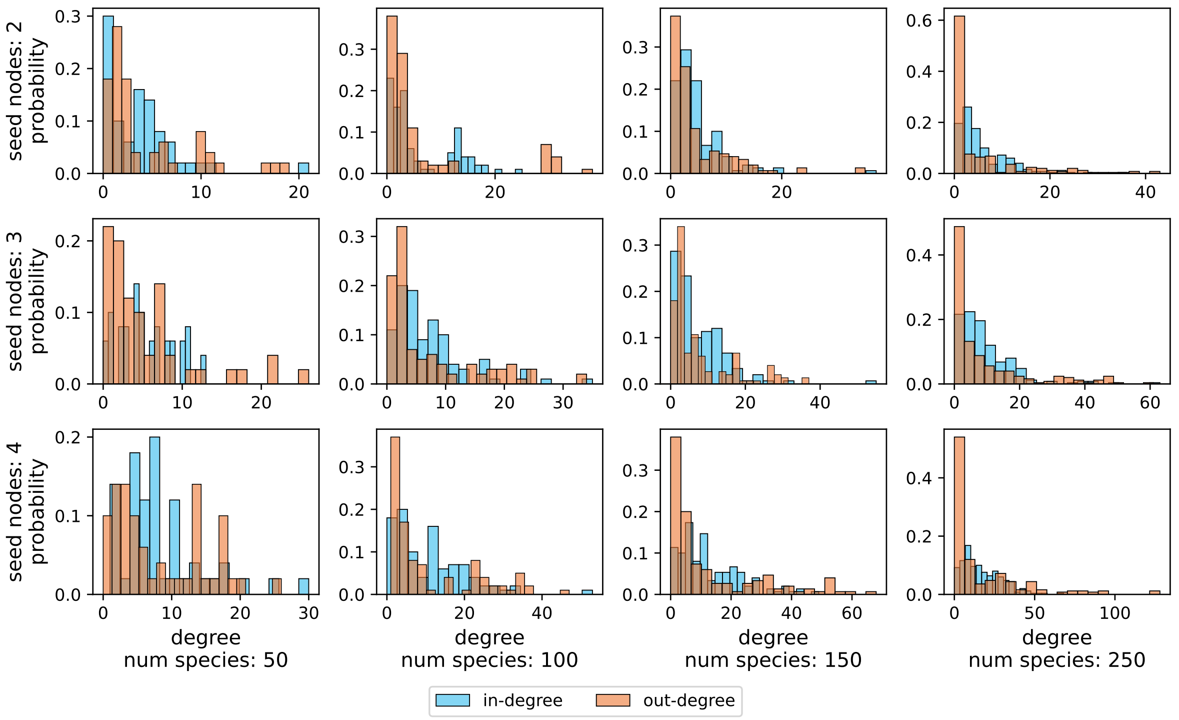

Figure S5: The figure reports the in- and out-degree distribution of the preprocessed bacteria interaction network with scalefree topology and $n_{neg} = 0.10$

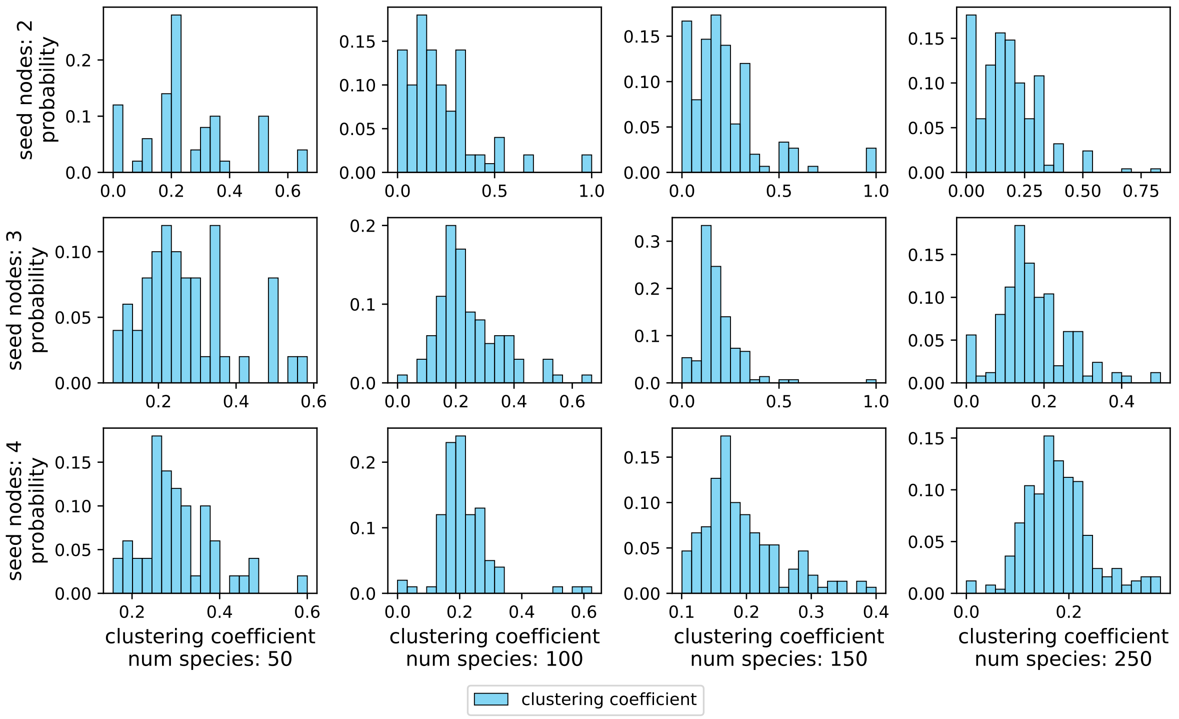

Figure S6: The figure reports the clustering coefficient distribution of the preprocessed bacteria interaction network with scalefree topology and $n_{neg} = 0.10$

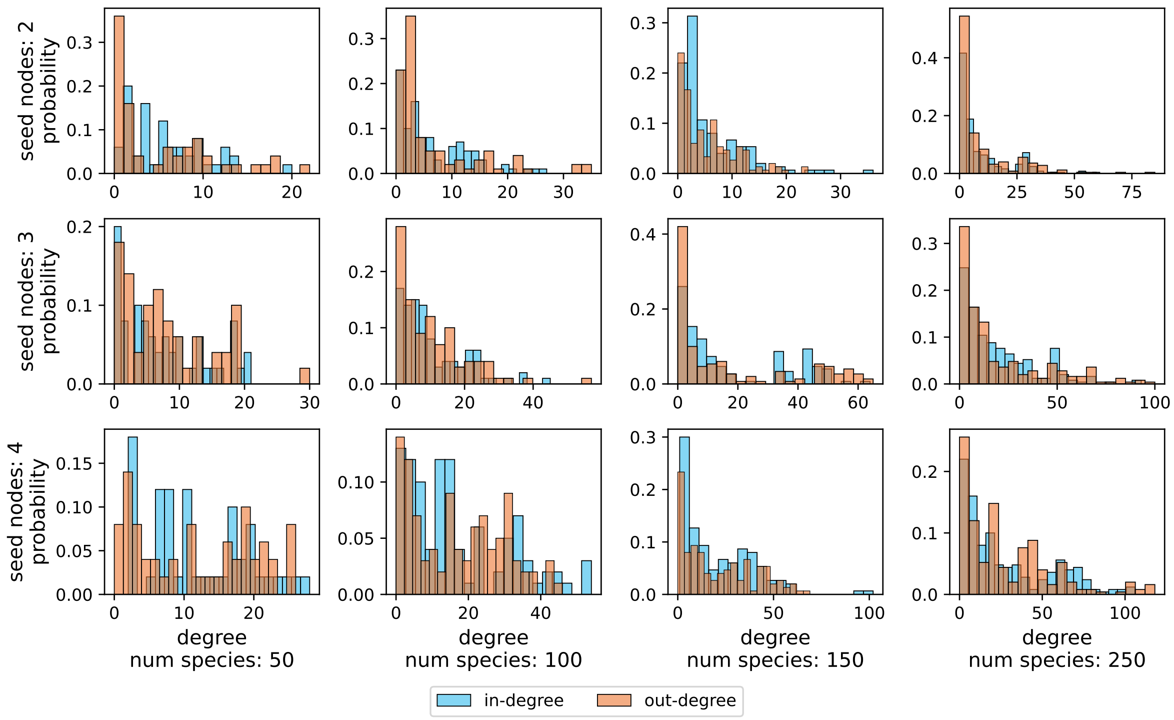

Figure S7: The figure reports the in- and out-degree distribution of the preprocessed bacteria interaction network with scalefree topology and $n_{neg} = 0.20$

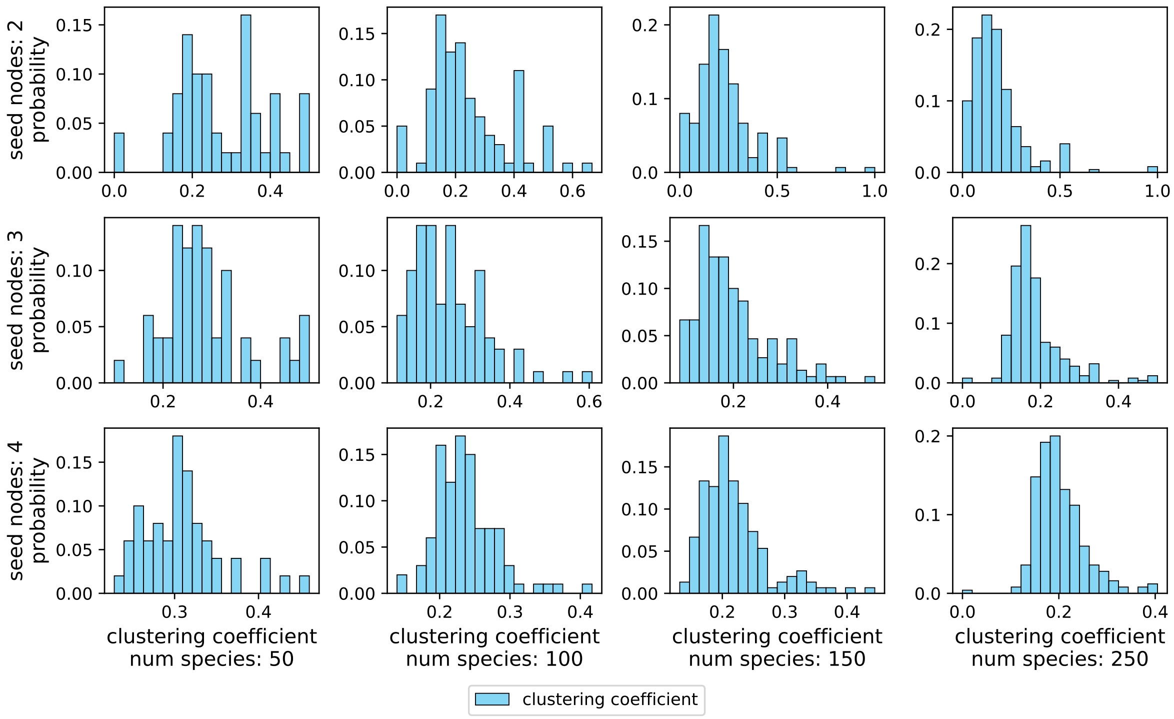

Figure S8: The figure reports the clustering coefficient distribution of the preprocessed bacteria interaction network with scalefree topology and $n_{neg} = 0.20$

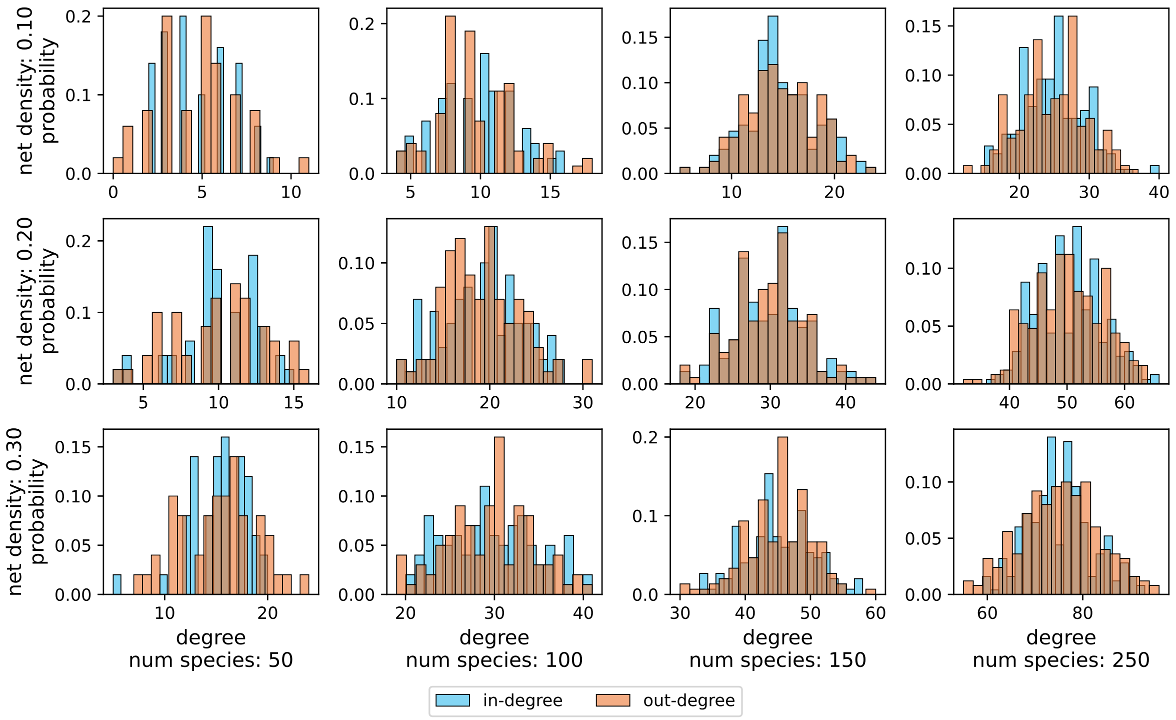

Figure S9: The figure reports the in- and out-degree distribution of the preprocessed bacteria interaction network with random topology and $n_{neg} = 0.00$

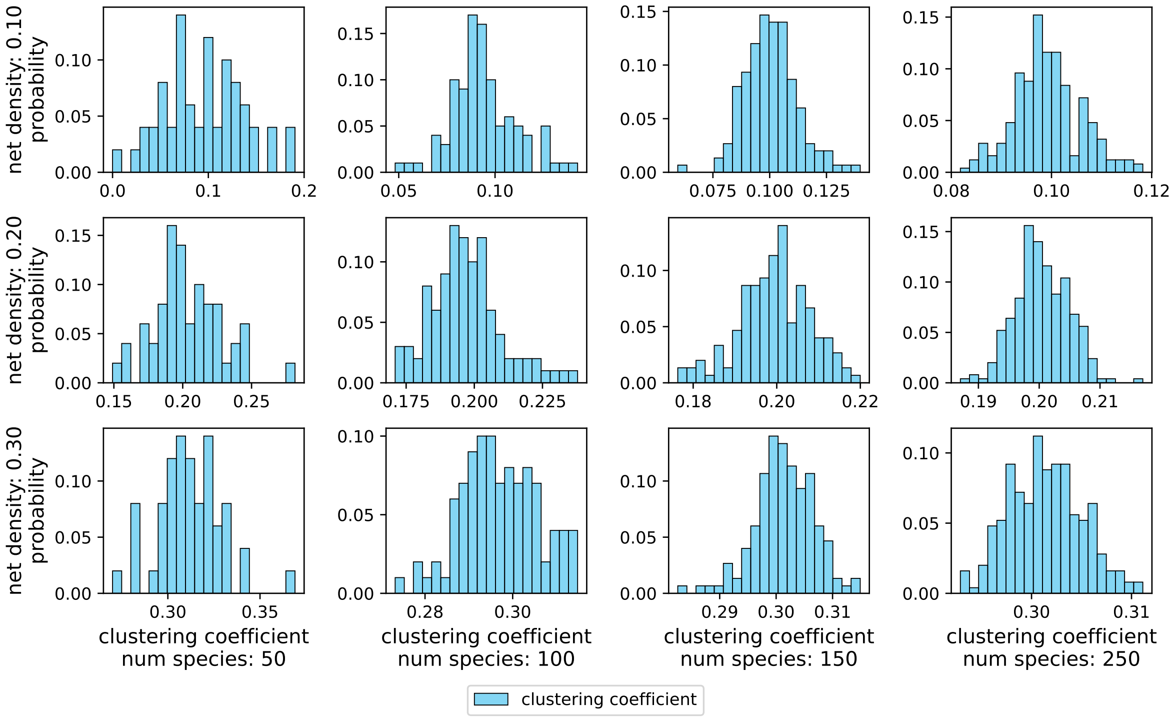

Figure S10: The figure reports the clustering coefficient distribution of the preprocessed bacteria interaction network with random topology and $n_{neg} = 0.01$

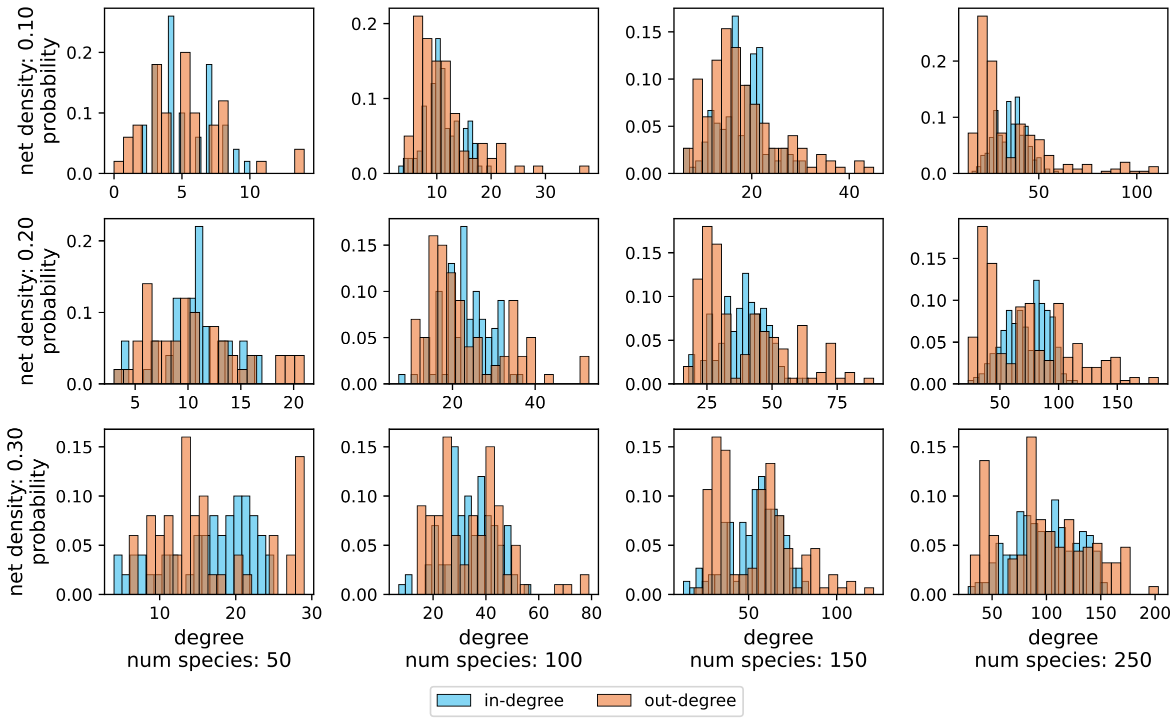

Figure S11: The figure reports the in- and out-degree distribution of the preprocessed bacteria interaction network with random topology and $n_{neg} = 0.01$

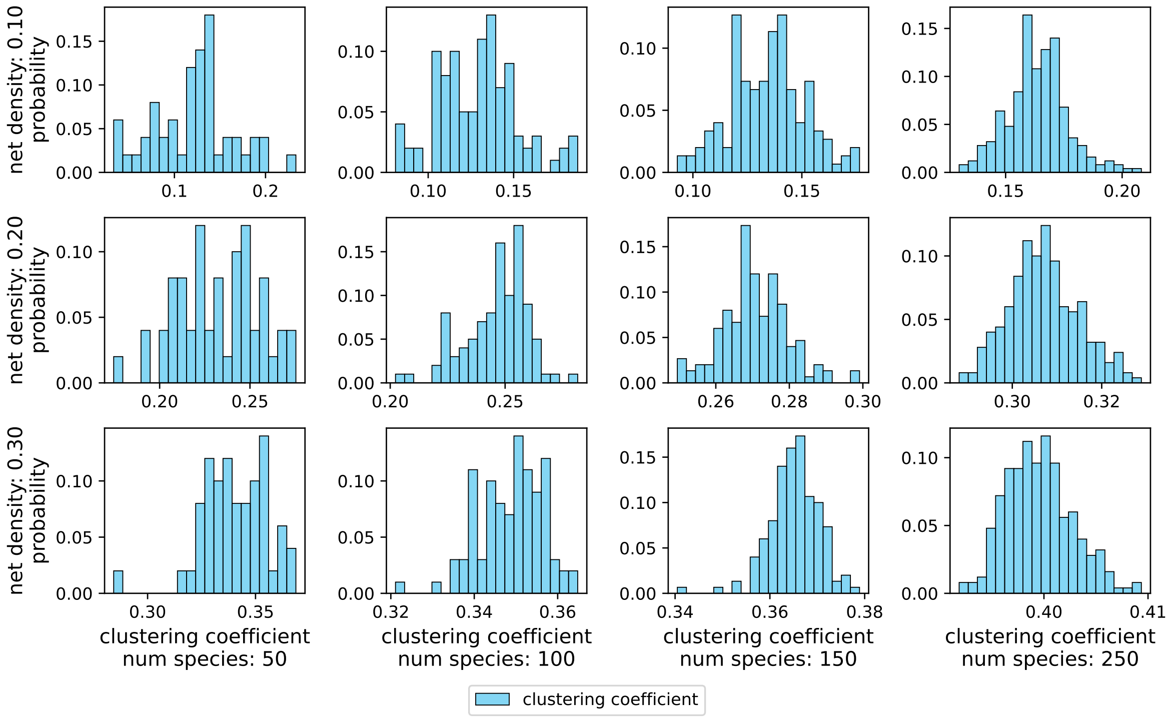

Figure S12: The figure reports the clustering coefficient distribution of the preprocessed bacteria interaction network with random topology and $n_{neg} = 0.01$

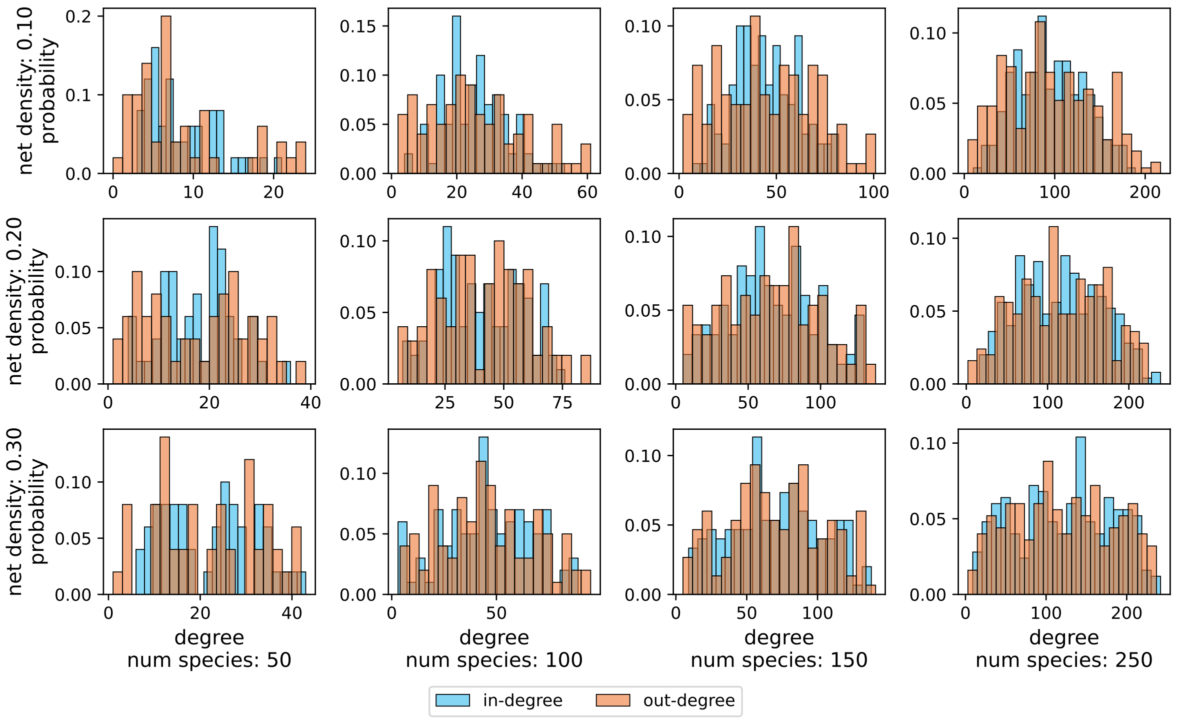

Figure S13: The figure reports the in- and out-degree distribution of the preprocessed bacteria interaction network with random topology and $n_{neg} = 0.10$

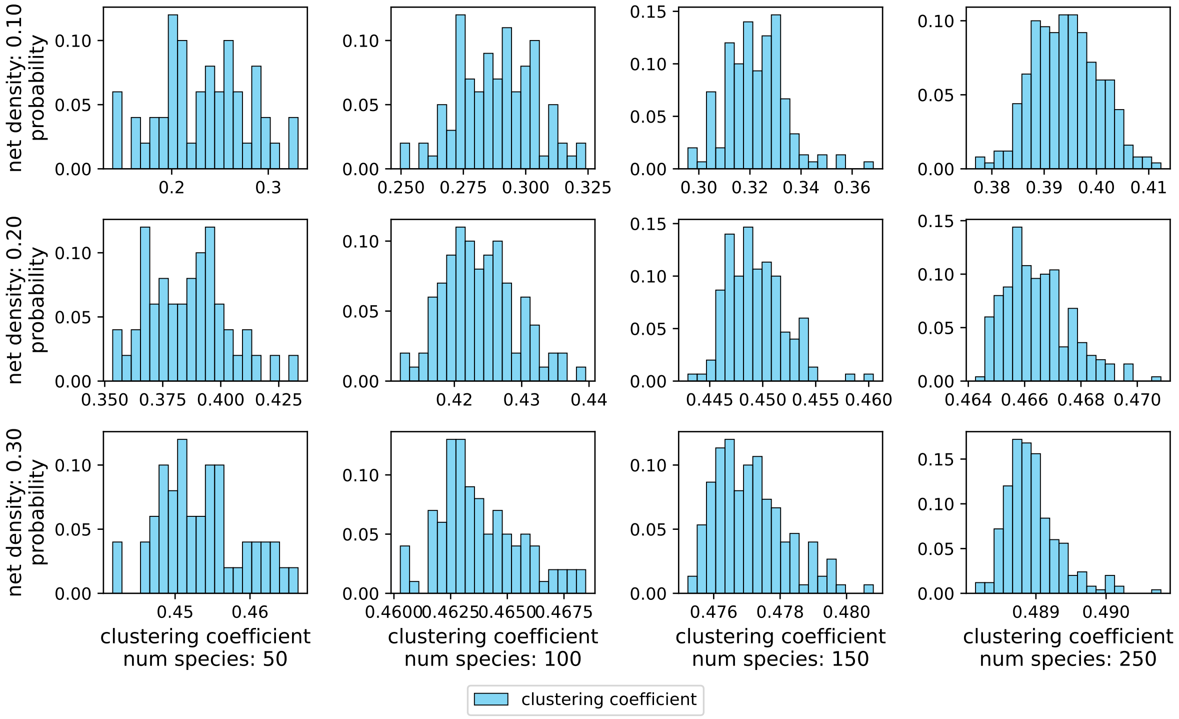

Figure S14: The figure reports the clustering coefficient distribution of the preprocessed bacteria interaction network with random topology and $n_{neg} = 0.10$

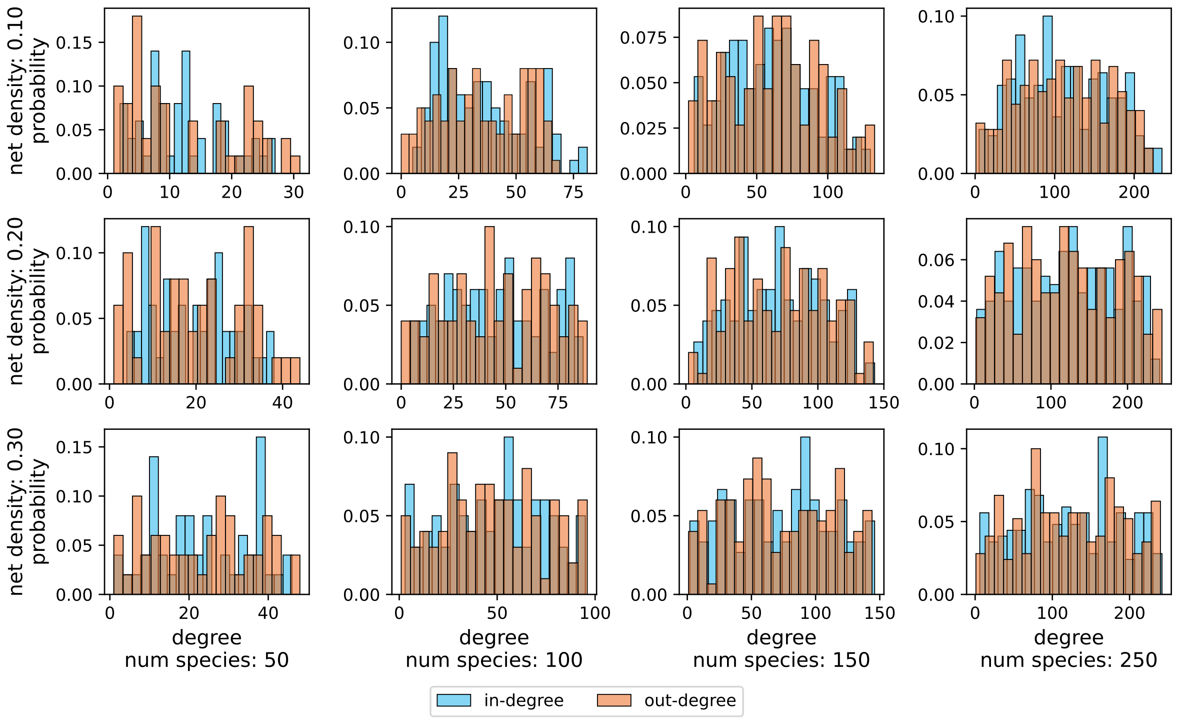

Figure S15: The figure reports the in- and out-degree distribution of the preprocessed bacteria interaction network with random topology and $n_{neg} = 0.20$

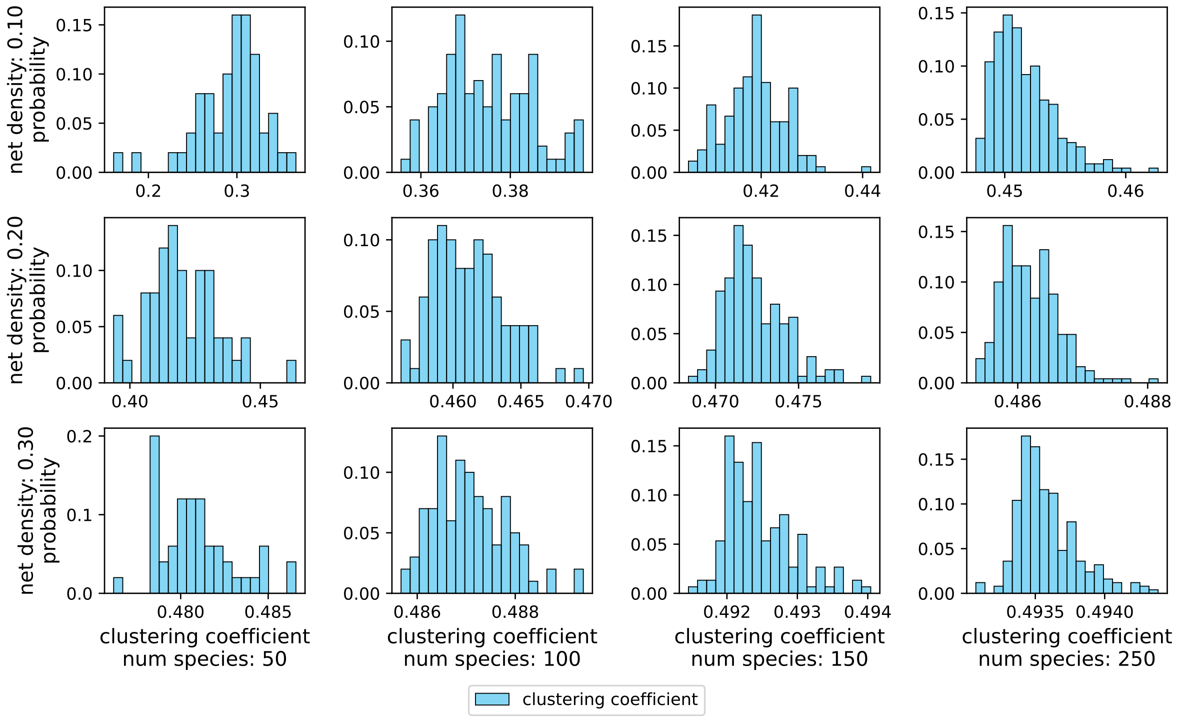

Figure S16: The figure reports the clustering coefficient distribution of the preprocessed bacteria interaction network with random topology and $n_{neg} = 0.20$

2.2 Effect of negative competitions on steady state distributions

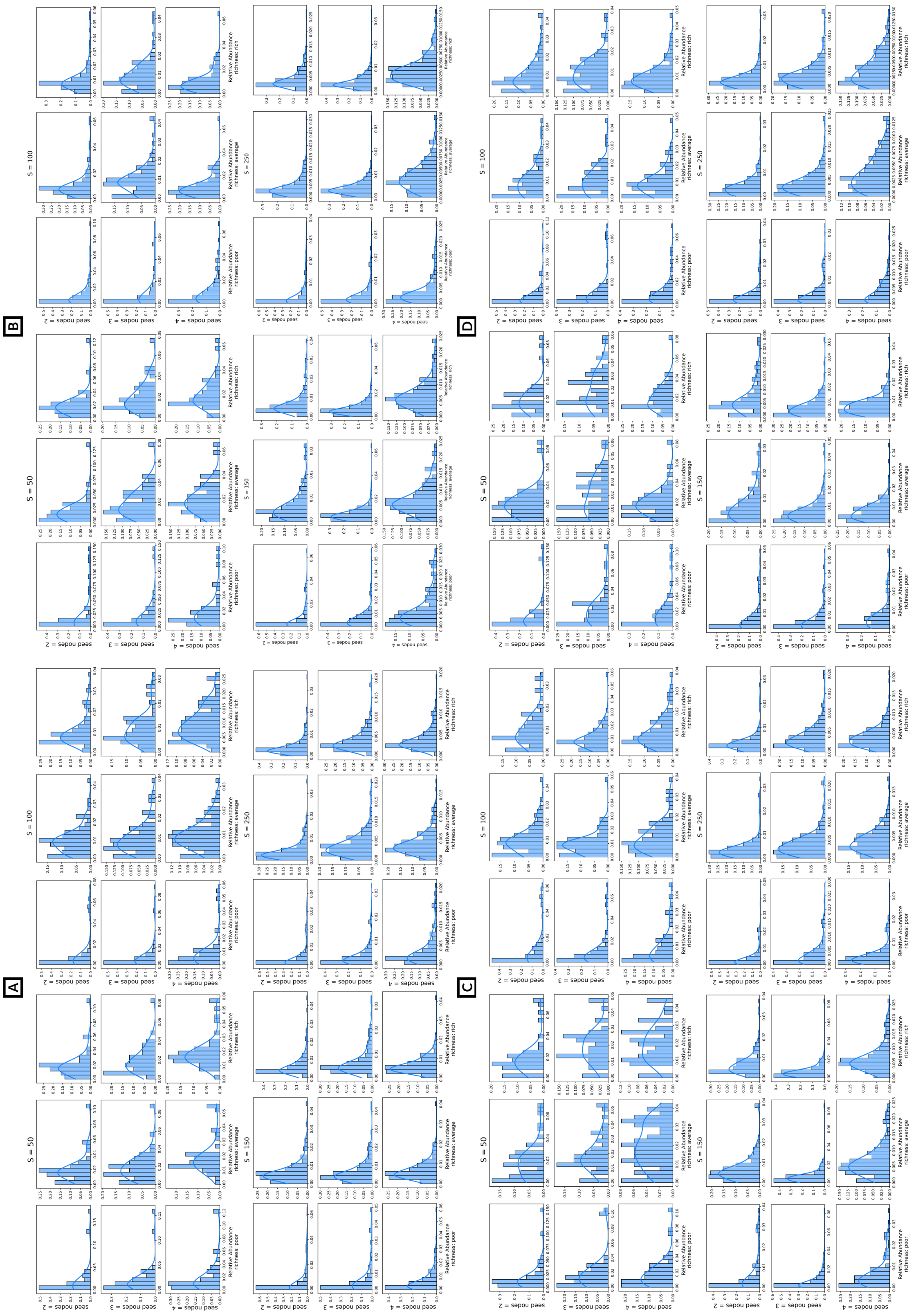

Figure S17: Effect of ratio of competitions over the steady state distributions of taxa abundances of a microbial community driven by a scalefree bacteria interaction network. Panels A), B), C) and D) are respectively related to a ratio of competitions of n_{neg} =0.05, 0.10, 0.15 and 0.20.

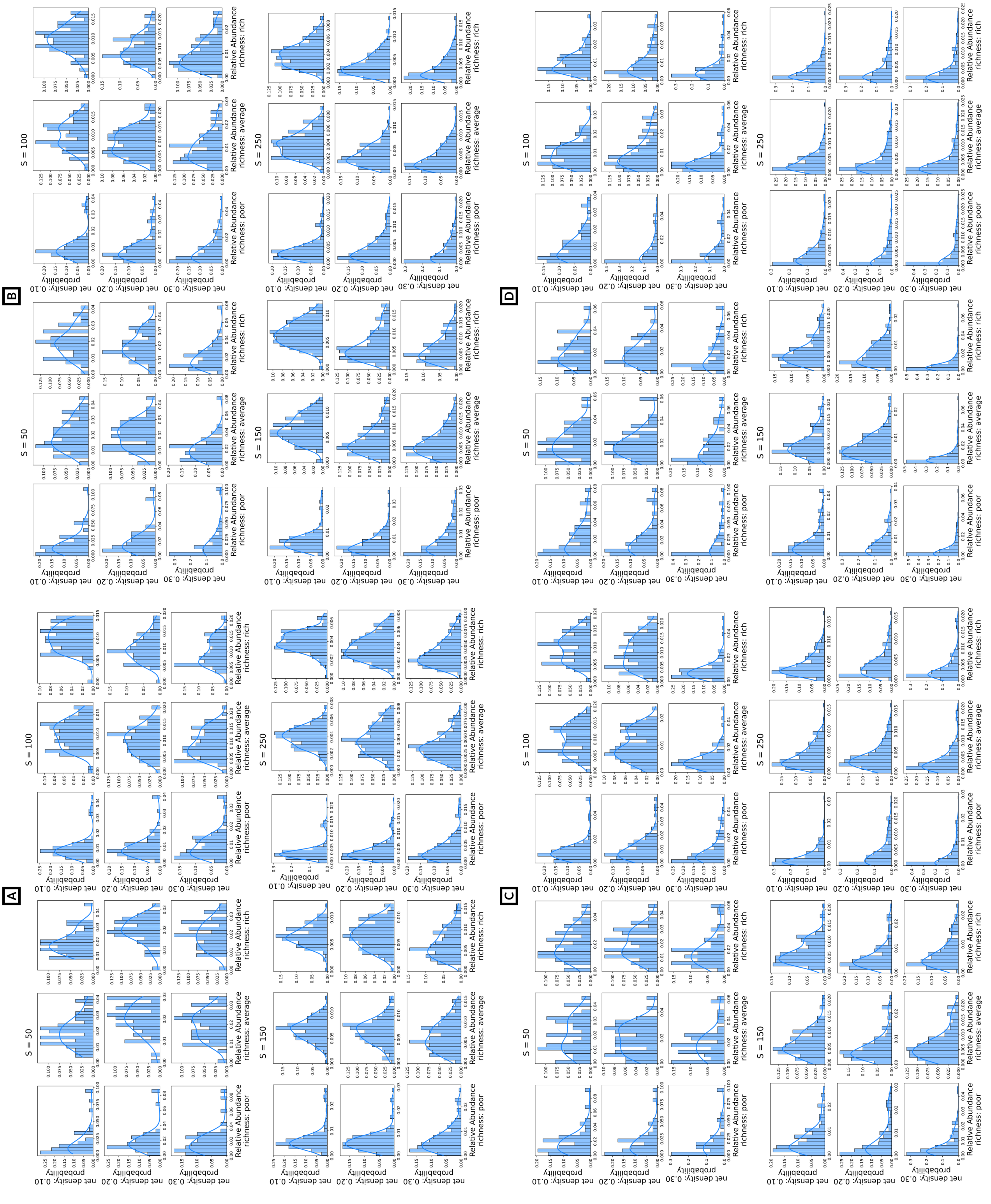

Figure S18: Effect of ratio of competitions over the steady state distributions of taxa abundances of a microbial community driven by a random bacteria interaction network. Panels A), B), C) and D) are respectively related to a ratio of competitions of n_{neg} = 0.05, 0.10, 0.15 and 0.20.

2.3 A small-scale comparison of network inference methods

3 TABLES

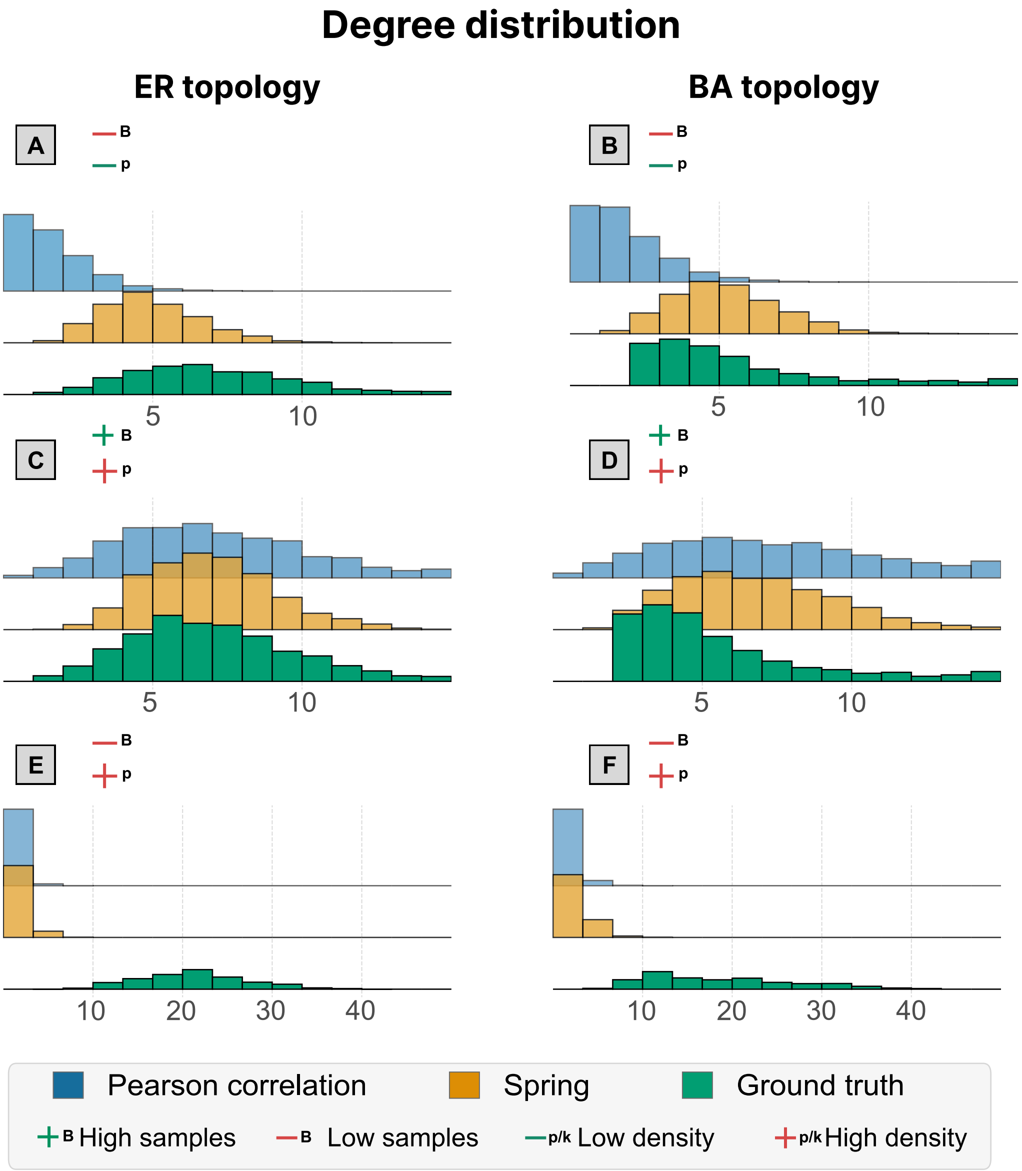

Figure S19: Degree distribution for the ER model (left) and BA model (right). In each panel the true distribution (green) is compared against the distributions obtained using Pearson correlation (blue) and SPRING (yellow). The first row (A and B) shows the results with simulation parameters $B = 100$ (#samples) and $p = 0.05,\ k = 2$ (density paramters). The second (C and D) row shows the results with simulation parameters $B = 300$ and $p = 0.15,\ k = 6$. The last row (E and F) shows the results with simulation parameters $B = 300$ and $p = 0.15,\ k = 6$.

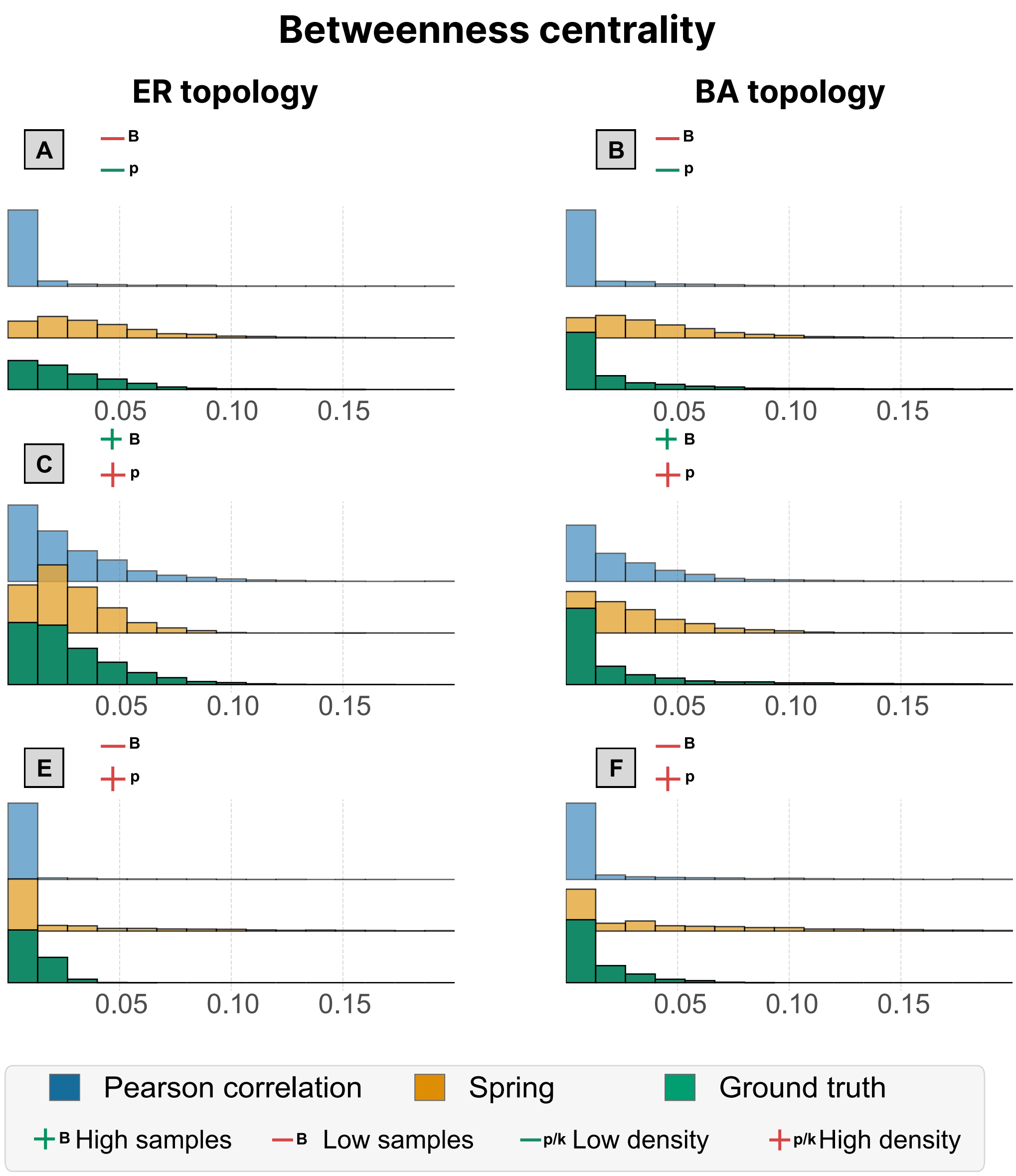

Figure S20: Betweenness centrality distribution for the ER model (left) and BA model (right). In each panel the true distribution (green) is compared against the distributions obtained using Pearson correlation (blue) and SPRING (yellow). The first row (A and B) shows the results with simulation parameters $B = 100$ (#samples) and $p = 0.05,\ k = 2$ (density paramters). The second row (C and D) shows the results with simulation parameters $B = 300$ and $p = 0.15,\ k = 6$. The last row (E and F) shows the results with simulation parameters $B = 300$ and $p = 0.15,\ k = 6$.

Table S1: Parameter tables with symbol, name, section of the procedure they are related to, short description, default value (when known), variable type, options (when available) and option parameters. Default is represented as value [unit of measurement].

Symbol	Class	Description	Default	Type	Options	Option parameters
S	Network generation	Number of taxa	50 [unitless]	int		
topology	Network generation	Bacteria interaction network topology		str	- random<br>- scalefree<br>- scalefree_bo	- {p}<br>- $\{k\}$<br>- $\{\alpha, \beta, \gamma, \delta_{in}, \delta_{out}\}\}$
n_{neg}	Network generation	Ratio of competitions	0.10 [unitless]	float		
unique	Network generation	Flag to preprocess the network to remove ambiguity	True [unitless]	bool		
directed	Network generation	Flag to generate a directed network	True [unitless]	bool		
consumer sampling	Community rules	Method to sample consumer preferences rows		str	- binary<br>- uniform<br>- gaussian<br>- gamma	- {}<br>- {}<br>- $\{\mu, \sigma\}$<br>- $\{k_g, \theta\}$
metabolic sampling	Community rules	Method to sample metabolic rules columns		str	- uniform<br>- dirichlet	- {}<br>- $\{\alpha_m\}$
N_0	Init microbial community	If known, initial abundances for each taxon		vec{float}		
R_0	Init microbial community	If known, initial resource concentration for each metabolite		vec{float}		
preset name	Init microbial community	Preset name defined in Patuzzi et al., 2019		str		
f_r	Init microbial community	Percentage of supplied resources		float		
food selection	Init microbial community	Method to select which resources to supply		str	- random	
R^0	Dynamics	Intrinsic equilibrium abundance for resources	1000 [mass/volume]	float		
m_c	Dynamics	Unitary metabolic cost	1 [energy/time]	float		
g	Dynamics	Conversion factor from energy uptake to growth rate	1 [1/energy]	float		
l	Dynamics	Leakage factor	0.8 [unitless]	float		
w	Dynamics	Energy content for resources	1 [energy/mass]	float		
τ	Dynamics	Timescale for externally supplied resource turnover	1 [time]	float		
r	Dynamics	Rate of resources self-renewal	1 [volume/mass/time]	float		
n_{neg}	Dynamics	Hill coefficient for metabolic regulation	10 [unitless]	float		
n_{hill}	Dynamics	Hill coefficient for functional response	2 [unitless]	float		
σ_{max}	Dynamics	Maximum input flux	1 [mass/time]	float		
supply	Dynamics	Method of how resources are supplied	external	str	- off<br>- external<br>- self-renewing	

Continued on next page

Table S1 – continued from previous page

Symbol	Class	Description	Default	Type	Options	Option parameters
regulation	Dynamics	Method of how the metabolic regulation occur		str	- independent<br>- energy<br>- mass	
response	Dynamics	Method of how the taxa respond functionally to input flux		str	- type I<br>- type II<br>- type III	
R_p	Init perturbed community	If known, perturbed resource concentration for each metabolite		vec{float}		
f_p	Init perturbed community	Percentage of perturbed resources	0.5 [unitless]	float		
p_s	Init perturbed community	Perturbation strength	2 [unitless]	float		
perturbation select method	Init perturbed community	Method to select perturbed resources		str	- random	
n cores	Numerical integration	Number of cores available for numerical integration of ODEs	1 [unitless]	int		
T	Numerical integration	Integration horizon	50 [time]	float		
n_s	Numerical integration	Number of time points	100 [unitless]	int		
libsize select method	Sequencing	Method to sample libsizes		str	- interpolate	

REFERENCES

Marsland, R., Cui, W., Goldford, J., and Mehta, P. (2020a). The community simulator: A python package for microbial ecology. *PLOS ONE* 15, 1–18. doi:10.1371/journal.pone.0230430

Marsland, R., Cui, W., Goldford, J., Sanchez, A., Korolev, K., and Mehta, P. (2019). Available energy fluxes drive a transition in the diversity, stability, and functional structure of microbial communities. *PLoS computational biology* 15, e1006793

Marsland, R., Cui, W., and Mehta, P. (2020b). A minimal model for microbial biodiversity can reproduce experimentally observed ecological patterns. *Scientific Reports* 10. doi:10.1038/s41598-020-60130-2

4 GENERATIVE AI DISCLOSURE

The authors acknowledge the use of Generative AI (OpenAI, GPT-4.0) to assist in editing and improving the accuracy and clarity of the English language in this work. The prompt that were used were the following:

> You are an academic and an expert in scientific communication. Assess and, where necessary, improve the clarity of the following paragraph: ””insert paragraph””. Make sure to maintain a consistent language register throughout.

The authors acknowledge that they revised the text produced by the generation model.
